# Mutant p53 promotes clonal hematopoiesis by generating a chronic inflammatory microenvironment

**DOI:** 10.1172/JCI184285

**Published:** 2025-12-30

**Authors:** Sisi Chen, Sergio Barajas, Sasidhar Vemula, Yuxia Yang, Ed Simpson, Hongyu Gao, Rudong Li, Farzaneh Behzadnia, Sarah C. Nabinger, David A. Schmitz, Hongxia Chen, Wenjie Cai, Shiyu Xiao, Ruyue Luo, Mohammed Abdullahel Amin, Maegan L. Capitano, James P. Ropa, Aidan Fahey, Shuyi Zhou, Tiffany M. Mays, Magdalena Sotelo, Hao Pan, Sophie K. Hu, Sophia Veranga, Moiez Ali, Maria Shumilina, Reuben Kapur, Kehan Ren, Yuzhi Jia, Huiping Liu, Irum Khan, Yasmin Abaza, Jessica K. Altman, Elizabeth A. Eklund, Lucy A. Godley, Christine R. Zhang, Peng Ji, Seth L. Masters, Ben A. Croker, H. Scott Boswell, George E. Sandusky, Zhonghua Gao, Lindsey D. Mayo, Sharon A. Savage, Stephanie Halene, Yali Dou, Leonidas C. Platanias, Madina Sukhanova, Yunlong Liu, Omar Abdel-Wahab, Yan Liu

**Affiliations:** 1 Precision Research Center for Refractory Diseases, Department of Hematology, Shanghai General Hospital, Shanghai Jiao Tong University School of Medicine, Shanghai, China.; 2 Molecular Pharmacology Program, Sloan Kettering Institute, Memorial Sloan Kettering Cancer Center, New York, New York, USA.; 3 Department of Medicine, Northwestern University Feinberg School of Medicine, Chicago, Illinois, USA.; 4 Department of Biochemistry and Molecular Biology, and; 5 Department of Pediatrics, Herman B. Wells Center for Pediatric Research, Indiana University School of Medicine, Indianapolis, Indiana, USA.; 6 Department of Medical Genetics, School of Basic Medical Sciences, Peking University, Beijing, China.; 7 Department of Medical Genetics, Indiana University School of Medicine, Indianapolis, Indiana, USA.; 8 Department of Hematology and Oncology, Chongqing University Three Gorges Hospital, Chongqing, China.; 9 Department of Microbiology and Immunology, Indiana University School of Medicine, Indianapolis, Indiana, USA.; 10 Department of Pathology and; 11 Department of Pharmacology, Northwestern University Feinberg School of Medicine, Chicago, Illinois, USA.; 12 Robert H. Lurie Comprehensive Cancer Center, Chicago, Illinois, USA.; 13 Department of Medicine, Jesse Brown VA Medical Center, Chicago, Illinois, USA.; 14 The Walter and Eliza Hall Institute of Medical Research, Parkville, Victoria, Australia.; 15 Department of Pediatrics, University of California San Diego, La Jolla, California, USA.; 16 Department of Medicine and; 17 Department of Pathology and Laboratory Medicine, Indiana University School of Medicine, Indianapolis, Indiana, USA.; 18 Department of Biochemistry and Molecular Biology, College of Medicine, Pennsylvania State University, Hershey, Pennsylvania, USA.; 19 Clinical Genetics Branch, Division of Cancer Epidemiology and Genetics, National Cancer Institute, Bethesda, Maryland, USA.; 20 Department of Internal Medicine and Yale Cancer Center, Yale University School of Medicine, New Haven, Connecticut, USA.; 21 Department of Medicine, University of Southern California, Los Angeles, California, USA.

**Keywords:** Hematology, Inflammation, Cytokines, Hematopoietic stem cells, NF-kappaB

## Abstract

Older individuals with somatic *TP53* mutations manifest clonal hematopoiesis (CH) and are at high risk of developing myeloid neoplasms. However, the underlying mechanisms are not fully understood. Here, we show that inflammatory stress confers a competitive advantage to p53 mutant hematopoietic stem and progenitor cells (HSPCs) by activating the NLRP1 inflammasome and increasing the secretion of pro-inflammatory cytokines such as IL-1β, inhibiting WT HSPC fitness in a paracrine fashion. During aging, mutant p53 dysregulates pre-mRNA splicing in HSPCs, leading to enhanced NF-κB activation and increased secretion of IL-1β and IL-6, thereby generating a chronic inflammatory bone marrow microenvironment. Furthermore, blocking IL-1β with IL-1β neutralizing antibody or inhibiting IL-1β secretion using gasdermin D inhibitor decreases the fitness of p53 mutant HSPCs. Thus, our findings uncover an important role for mutant p53 in regulating inflammatory signaling in CH and suggest that curbing inflammation may prevent the progression of *TP53*-mutant CH to myeloid neoplasms.

## Abstract 2

We report that mutant p53 increases chromatin accessibility to the NLRP1 inflammasome and dysregulates pre-mRNA splicing in key regulators of NF-κB in HSPCs, thereby generating a chronic inflammatory microenvironment to promote clonal hematopoiesis.

## Graphical abstract

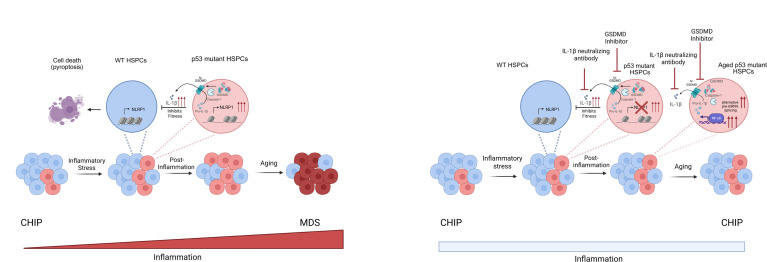

## Introduction

Human aging is associated with increased occurrence of clonal hematopoiesis (CH) both with normal blood counts (CH of indeterminate potential [CHIP]) and with clonal cytopenias of unknown significance (CCUS), where mutated hematopoietic stem and progenitor cells (HSPCs) undergo clonal expansion in older individuals without evidence of frank neoplasia (1–4). CH is associated with increased risks of developing myelodysplastic syndromes (MDSs) and acute myeloid leukemia (AML) (5–7). However, the underlying mechanisms are not fully understood.

Chronic inflammation has been implicated in cancer initiation, progression, and metastasis (8). Chronic and low-grade inflammation develops during aging (9, 10), which may contribute to the pathogenesis of CH and MDS (11, 12). Indeed, some older individuals with CH display an aberrant systematic inflammatory milieu compared with older individuals without CH (13, 14). Inflammatory stress has been implicated in the expansion of *Tet2*, *Dnmt3a*, and *Asxl1* mutated HSPCs in animal models of CH (15–17). However, whether chronic inflammation promotes the progression of CH to myeloid neoplasms is largely unexplored.

The tumor suppressor gene *TP53* is frequently mutated in human cancer (18, 19). Acquired somatic gain-of-function mutant p53 proteins, such as p53^R248W^ and p53^Y220C^, were identified in patients with CH (5–7). We and others discovered that genotoxic stresses, including radiation and chemotherapy, promote *TP53*-mutant HSPC expansion (20–23). Mechanistically, mutant p53 drives CH through modulating epigenetic pathways (24). Somatic *TP53* mutations in CH are associated with an increased incidence of MDS and AML (5–7). We found that mutant p53 promotes leukemia development through enhancing leukemia-initiating cell self-renewal (25). *TP53* mutations are present in 10%–15% of primary and in 20%–30% of secondary MDS cases (26, 27). CH and, in particular, MDS are frequently caused by mutations in key factors of the spliceosome, including *SF3B1*, *SRSF2*, *U2AF1*, and *ZRSR2* (28, 29). Altered splicing has been implicated in pathogenesis of hematological malignancies (28–30). Notably, both older human and mouse HSPCs display dysregulated pre-mRNA splicing (31–33). However, whether mutant p53 alters pre-mRNA splicing in HSPCs and drives MDS development is not known.

In this study, we report that mutant p53 increases chromatin accessibility to the *NLRP1* inflammasome gene and dysregulates pre-mRNA splicing in key regulators of NF-κB in HSPCs, thereby generating a chronic inflammatory microenvironment to promote CH.

## Results

### Inflammatory stress confers a competitive advantage to p53 mutant HSPCs.

**Figure 1:**
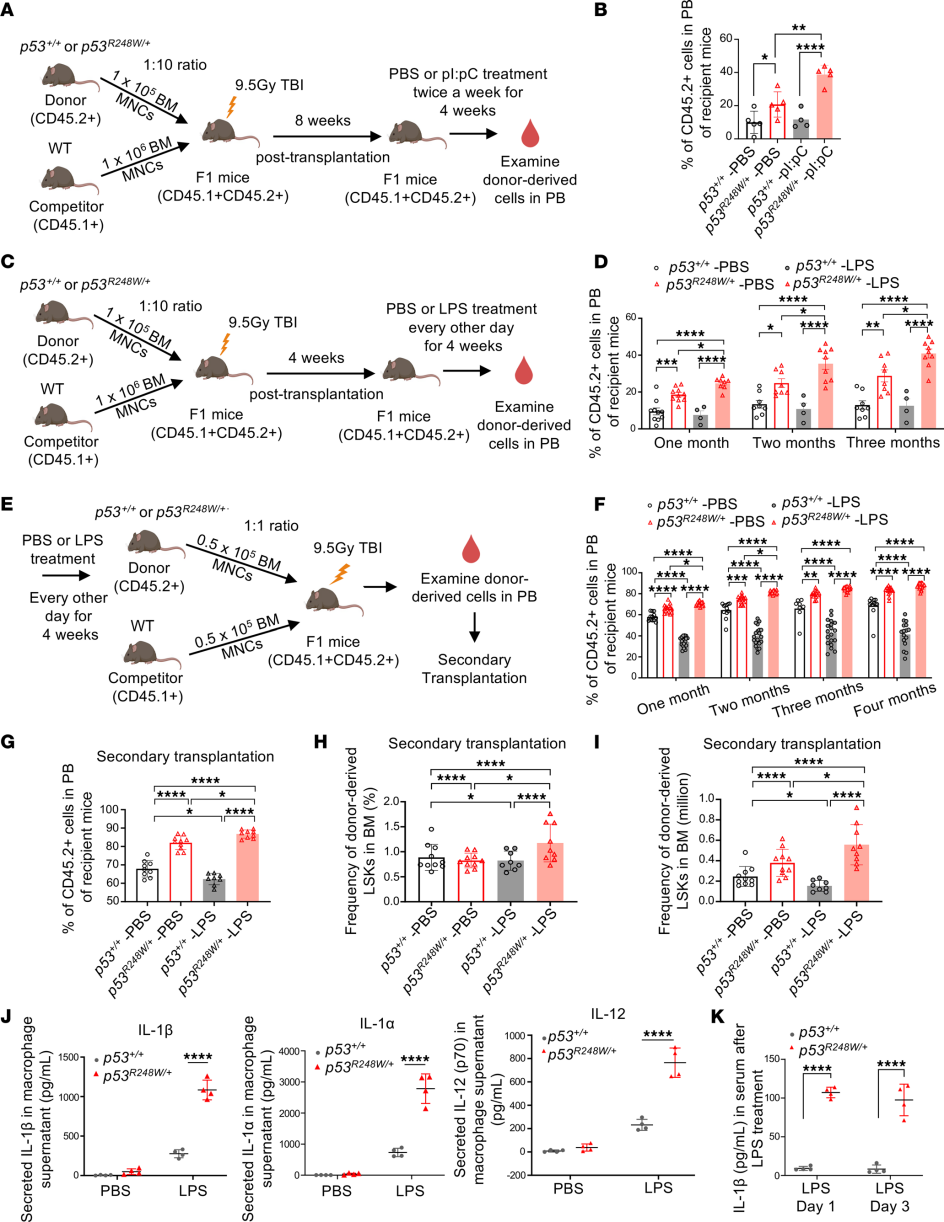
Inflammatory stress confers a competitive advantage to p53 mutant HSPCs. (**A**) Schematic illustration of experimental design where recipient mice underwent competitive transplantation and were treated with either PBS or pI:pC (25 μg/kg, i.p.). MNC, mononuclear cell; TBI, total body irradiation. (**B**) Percentage of donor-derived cells (CD45.2^+^) in PB of recipient mice at 16 weeks following PBS or pI:pC treatment; *n* = 5 mice per group. (**C**) Schematic illustration of experimental design where recipient mice underwent competitive transplantation and were treated with PBS or LPS (1 mg/kg, i.p.). (**D**) Percentage of donor-derived cells in PB of recipient mice following PBS or LPS treatment; *n* = 6–8 mice per group. (**E**) Schematic illustration of experimental design where BM from primary mice treated with PBS or LPS (1 mg/kg, i.p.) were used for competitive transplantation. (**F**) Percentage of donor-derived cells in PB of recipient mice; n = 10–15 mice per group. (**G**) The frequency of donor-derived cells in PB of secondary recipient mice; *n* = 9 mice per group. (**H**) The frequency of donor-derived LSKs in the BM of secondary recipient mice; *n* = 9 mice per group. (**I**) The number of donor-derived LSKs in the BM of secondary recipient mice; *n* = 9 mice per group. (**J**) *p53^+/+^* and *p53^R248W/+^* macrophages were stimulated with LPS (100 ng/mL), and the levels of secreted cytokines and chemokines were assayed using a mouse 31-Plex Cytokine/Chemokine array; *n* = 4 biological replicates. (**K**) *p53^+/+^* and *p53^R248W/+^* mice were treated with LPS (1 mg/kg, i.p.), and PB serum was collected on days 1 and 3 after LPS treatment. The levels of cytokines and chemokines were assayed by Cytokine/Chemokine array; *n* = 4 mice per group. The comparison among multiple groups was evaluated by 1-way ANOVA (**B** and **G**–**I**) or 2-way ANOVA (**D** and **F**). The comparison between 2 groups was evaluated by 2-tailed *t* test (**J** and **K**). Statistical significance: *P < 0.05, **P < 0.01, ***P < 0.001, ****P < 0.0001.

Both viral and bacterial infections activate inflammatory response and induce chronic inflammation (8). The double-stranded RNA mimetic polyinosinic-polycytidylic acid (pI:pC) mimics the viral genome of many RNA viruses, and prolonged pI:pC treatment induces chronic inflammation in mice (34). To determine whether viral infection expands p53 mutant HSPCs in vivo, we performed competitive bone marrow (BM) transplantation assays. Eight weeks following transplantation, recipient mice were treated with PBS or pI:pC twice a week for 4 weeks (Figure 1A). While pI:pC treatment had no effect on the engraftment of WT BM cells, it significantly increased the engraftment of p53 mutant cells in peripheral blood (PB) of the recipient mice compared with those treated with PBS (Figure 1B). Given that pI:pC induces DNA damage in hematopoietic cells (34) and the canonical function of p53 is a mediator of DNA damage response (18, 19), we examined γH2AX in PBS- or pI:pC-treated Lin^−^ BM cells. p53 mutant HSPCs displayed more γH2AX foci compared with WT HSPCs at steady state. However, pI:pC treatment increased the number of γH2AX foci in WT HPSCs but decreased H2AX phosphorylation in mutant cells (Supplemental Figure 1, A and B; supplemental material available online with this article; https://doi.org/10.1172/JCI184285DS1), suggesting that pI:pC stimulation enhances DNA damage repair in mutant HSPCs.

LPS is a ligand for TLR4, and LPS treatment activates inflammatory response in hematopoietic cells, promoting HSPC proliferation while reducing competitive fitness (35). To determine the impact of LPS on p53 mutant HSPCs, we performed competitive BM transplantation assays to determine whether LPS treatment expands p53 mutant HSPCs in vivo. Four weeks following transplantation, recipient mice were treated with LPS or PBS every other day for 4 weeks (Figure 1C). We found that LPS treatment significantly increased the engraftment of *p53^R248W/+^* cells in PB of recipient mice compared with those treated with PBS (Figure 1D).

To determine the impact of LPS on HSPC function in vivo, we treated *p53^+/+^* and *p53^R248W/+^* mice with LPS or PBS every other day for a month. We then isolated live BM cells from LPS- or PBS-treated donor mice and performed competitive transplantation assays (Figure 1E). Consistent with a previous report (35), LPS-treated WT BM cells showed decreased engraftment in PB and BM in both primary and secondary transplantation assays, whereas LPS-treated p53 mutant BM cells continued to show increased engraftment (Figure 1, F and G, and Supplemental Figure 1, C and D). Importantly, LPS treatment increased both the frequency and absolute number of p53 mutant LSKs in the BM of secondary recipient mice compared with PBS treatment (Figure 1, H and I).

To understand how LPS-induced inflammatory stress promotes p53 mutant HSPC expansion, we examined the impact of mutant p53 on cytokine secretion from BM-derived macrophages (36). We treated *p53^+/+^* and *p53^R248W/+^* macrophages with 100 ng/mL LPS for 12 h to induce pro-inflammatory activation. Supernatants were then collected and subjected to cytokine array analysis. Notably, p53 mutant macrophages showed increased secretion of multiple pro-inflammatory cytokines, including IL-1β, IL-1α, IL-12, KC, and CXCL-9, compared with WT macrophages following LPS stimulation (Figure 1J and Supplemental Figure 1E). Next, we treated *p53^+/+^* and *p53^R248W/+^* mice with 1 dose of LPS and examined cytokine and chemokine in PB serum of mice on day 1 (24 h) and day 3 (72 h) after LPS treatment. We observed increased levels of IL-1β in PB serum of *p53^R248W/+^* mice compared with that of the *p53^+/+^* mice on both days 1 and 3 after LPS treatment (Figure 1K). Thus, we demonstrated that mutant p53 enhances LPS-induced inflammatory response.

### p53 mutant HSPCs show enhanced NLRP1 activation.

**Figure 2:**
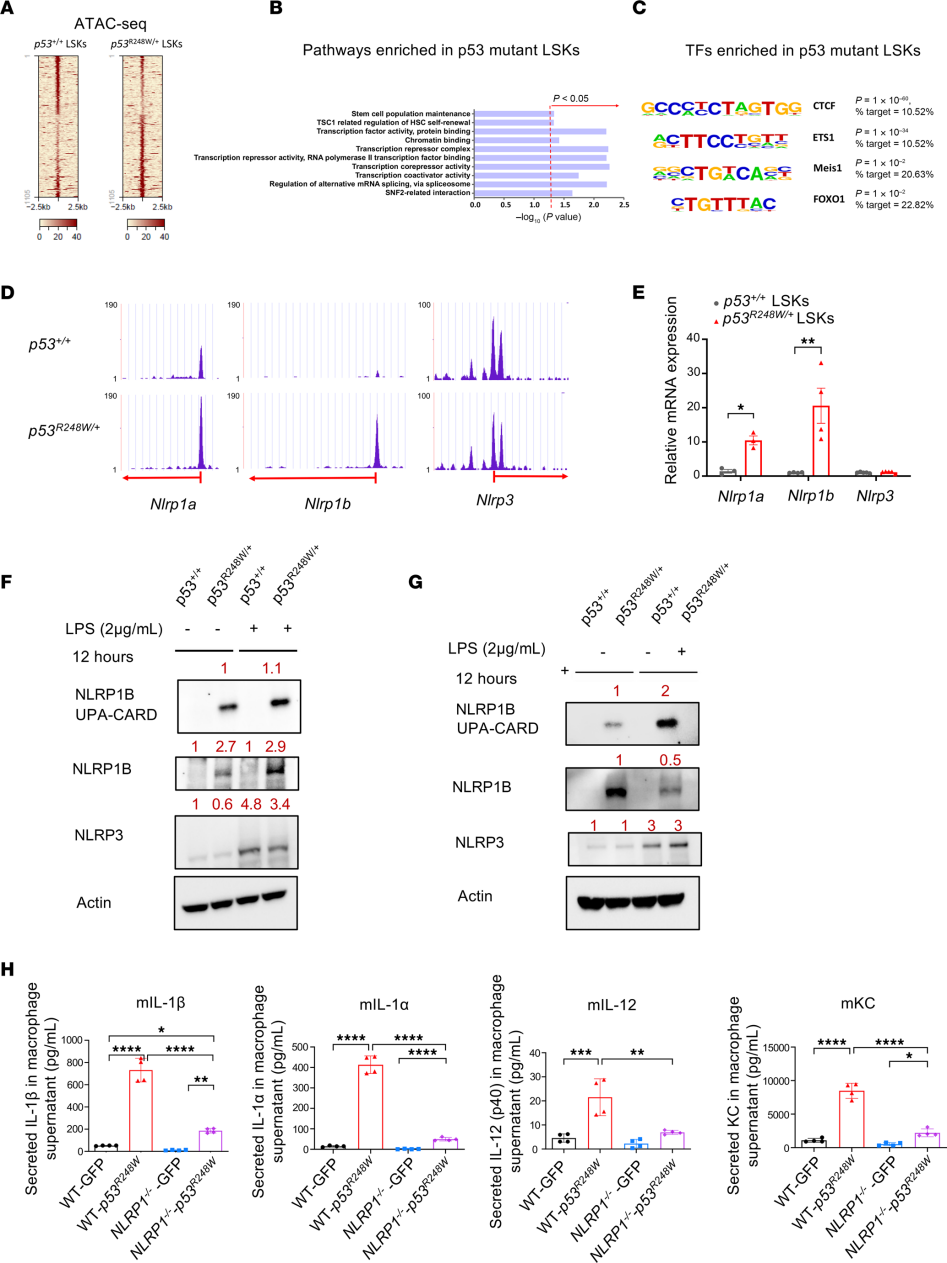
p53 mutant HSPCs show increased NLRP1 activation. (**A**) LSK cells were subjected to ATAC-seq assays to identify differential regions of chromatin accessibility. Heatmap shows differential active regions in *p53^+/+^* and *p53^R248W/+^* LSKs. (**B**) Enriched biological processes of genes associated with significantly upregulated ATAC-seq peaks in *p53^R248W/+^* LSKs analyzed by DAVID Functional Annotation Tool. (**C**) Enrichment of transcription factor (TF) motifs in peaks that gain open accessibility in *p53^R248W/+^* LSKs compared with *p53^+/+^* LSKs determined by HOMER de novo Known Motif Analysis. (**D**) *Nlrp1a* and *Nlrp1b* acquired significant open chromatin peaks in *p53^R248W/+^* LSKs. (**E**) *Nlrp1a* and *Nlrp1b* were significantly upregulated in *p53^R248W/+^* LSKs compared with *p53^+/+^* LSKs; *n* = 3 biological replicates. (**F**) The level of Nlrp1b was increased in *p53^R248W/+^* Lin^−^ cells. (**G**) The levels of Nlrp1b and Nlrp3 in BM-derived macrophages following LPS stimulation were determined by immunoblotting. (**H**) GFP or p53^R248W^ was introduced into *Nlrp1^+/+^* and *Nlrp1*^−*/*−^ Lin^−^ BM cells using retrovirus. Transduced cells (GFP^+^) were differentiated into macrophages. Macrophages were stimulated with LPS (100 ng/mL) for 12 hours, and the levels of secreted cytokines and chemokines were assayed using a Cytokine/Chemokine array; *n* = 4 biological replicates. The comparison among multiple groups was evaluated with 1-way ANOVA (**E** and **H**). Statistical significance: *P < 0.05, **P < 0.01, ***P < 0.001, ****P < 0.0001.

To investigate how mutant p53 regulates inflammatory response in HSPCs, we performed ATAC-seq (assay for transposase-accessible chromatin with sequencing) assays in LSK cells to identify differential regions of chromatin accessibility (37). There were approximately 600 peaks significantly upregulated in *p53^R248W/+^* LSKs compared with *p53^+/+^* LSKs (Figure 2A). Furthermore, unbiased DAVID (Database for Annotation, Visualization, and Integrated Discovery) pathway analysis of ATAC peaks significantly upregulated in *p53^R248W/+^* LSKs revealed enrichment in hematopoietic stem cell (HSC) maintenance and self-renewal pathways (Figure 2B) (38). On the contrary, the p53 pathway, canonical Wnt signaling, and negative regulation of immune system process were enriched in *p53^+/+^* LSKs (Supplemental Figure 2A). To characterize chromatin regions with differential accessibility in *p53^+/+^* and *p53^R248W/+^* LSKs, we performed a motif search of the gained openly accessible peaks in *p53^R248W/+^* LSKs (39). Motif analysis identified a strong enrichment for transcription factor binding sites, including CTCF, ETS1, MEIS1, and FOXO1, in *p53^R248W/+^* LSKs (Figure 2C), some of which are known regulators of HSPCs.

Mutant p53 increases chromatin accessibility to chemokine gene *Cxcl9* (Supplemental Figure 2B). Indeed, *Cxcl9* is upregulated in p53 mutant HSPCs (Supplemental Figure 2C). Notably, mutant p53 increases the chromatin accessibility to inflammasome genes, including *Nlrp1a* and *Nlrp1b* (Figure 2D), and we confirmed the upregulation of both *Nlrp1a* and *Nlrp1b* in *p53^R248W/+^* LSKs compared with *p53^+/+^* LSKs (Figure 2E). While NLRP3 inflammasome is activated in HSPCs from low- to high-risk MDS (40), mutant p53 affects neither the chromatin accessibility nor the expression of *Nlrp3* in LSKs (Figure 2, D and E). Inflammasomes are multiprotein complexes that activate caspase-1 and increase the release of pro-inflammatory cytokines such as IL-1β, leading to caspase-1–dependent death, known as pyroptosis (41). NLRP1 was one of the first inflammasome-forming pattern recognition receptors to be identified (42, 43). To examine inflammasome activation in HSPCs, we treated *p53^+/+^* and *p53^R248W/+^* Lin^−^ cells with LPS and performed immunoblotting analysis to examine the levels of Nlrp1b and Nlrp3. We observed increased levels of NLRP3 and active NLRP1B (NLRP1B-UPA-CARD) in mutant HSPCs at baseline and following LPS stimulation, whereas LPS stimulation increased NLRP3 levels in both *p53^+/+^* and *p53^R248W/+^* HSPCs (Figure 2F). In addition, we found increased levels of NLRP1B-UPA-CARD in mutant macrophages compared with WT cells (Figure 2G). Consistent with our findings in HSPCs, LPS stimulation increased NLRP3 levels in both *p53^+/+^* and *p53^R248W/+^* macrophages (Figure 2G). LPS stimulation also increased levels of cleaved caspase-1 in mutant macrophages compared with WT cells, whereas LPS increased levels of mature gasdermin D (GSDMD-NT) and cleaved caspase-3 in both *p53^+/+^* and *p53^R248W/+^* HSPCs (Supplemental Figure 2D). NLRP1 is known to sense and be activated by dsRNA (42). To determine the involvement of the cGAS/STING pathway in NLRP1 activation in mutant cells, we treated *p53^+/+^* and *p53^R248W/+^* Lin^−^ cells with pI:pC and examined levels of cGas and Sting. pI:pC stimulation increased the levels of cGas and Sting but decreased levels of p-Sting in both *p53^+/+^* and *p53^R248W/+^* cells (Supplemental Figure 2E).

To determine the contribution of NLRP1 to the clonal advantage of p53 mutant HSPCs, we crossed *Nlrp1*^−*/*−^ mice with *p53^R248W/+^* mice. As *Nlrp1* and *Trp53* are close to each other on mouse chromosome 11, we failed to generate *Nlrp1*^−*/*−^ *p53^R248W/+^* mice (43). We then ectopically expressed GFP or p53^R248W^ in WT and *Nlrp1*^−*/*−^ Lin^−^ BM cells using retroviruses. Transduced cells (GFP^+^) were differentiated into macrophages and stimulated with LPS for 12 h. Macrophage supernatants were then subjected to multiplex cytokine analysis. Loss of *Nlrp1* significantly decreased the secretion of multiple pro-inflammatory cytokines, including IL-1β, IL-1α, IL-12, and KC, from p53 mutant macrophages (Figure 2H). Thus, we demonstrated that p53 mutant HSPCs show enhanced NLRP1 activation and that NLRP1 is a key mediator of mutant p53-driven inflammation in hematopoietic cells.

### Resistance to inflammatory stress underlies enhanced fitness seen in p53 mutant HSPCs.

**Figure 3:**
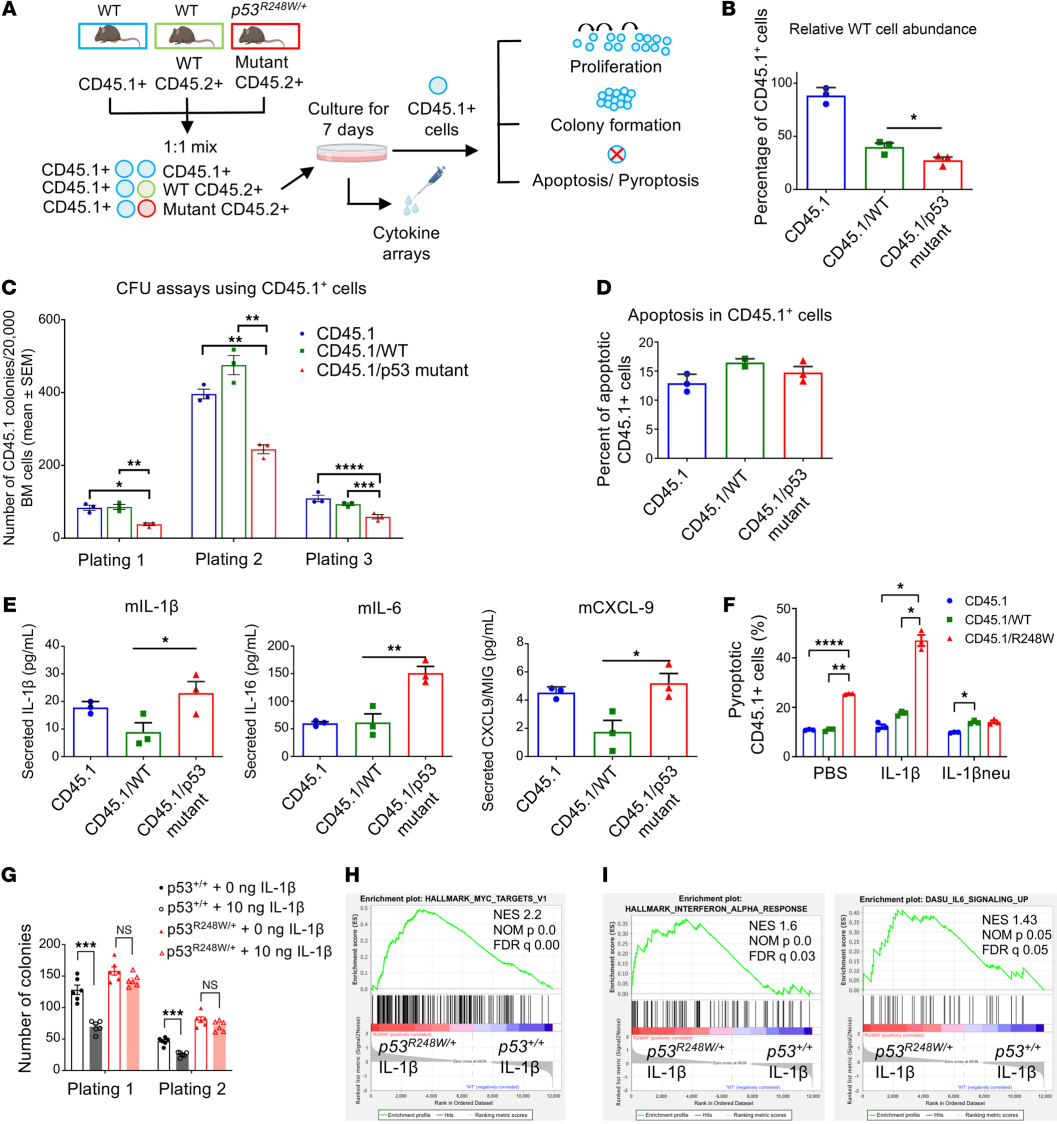
Resistance to inflammatory stress underlies enhanced fitness seen in p53 mutant HSPCs. (**A**) Schematic of in vitro cell competition assays using BM cells from *p53^+/+^* and *p53^R248W/+^* mice (CD45.2^+^) and WT competitor cells (CD45.1^+^). Three groups of BM cells, CD45.1, CD45.1/WT, and CD45.1/p53 mutant, were cultured in serum-free medium supplemented with SCF, TPO, FLT3 ligand, IL-3, and GM-CSF. 7 days later, live CD45.1^+^ cells from each group were sorted and used for proliferation, serial replating, apoptosis, and pyroptosis assays. In addition, cytokines and chemokines in the conditioned media were assayed by Cytokine/Chemokine array. (**B**) Proliferation of live CD45.1^+^ cells from 3 cell culture groups shown in **A**; *n* = 3 independent experiments performed in duplicate. (**C**) Serial replating assays of live CD45.1^+^ cells purified from 3 cell culture groups shown in **A**; *n* = 3 independent experiments performed in duplicate. (**D**) CD45.1^+^ cells purified from 3 cell culture groups were assayed for apoptosis; *n* = 3 independent experiments. (**E**) Increased levels of IL-1β, IL-6, and CXCL-9 were observed in the conditioned media from the CD45.1/p53 mutant group compared with the CD45.1/WT cell group; *n* = 3 independent experiments performed in duplicate. (**F**) Pyroptosis assays of CD45.1^+^ cells purified from 3 cell culture groups in the presence of PBS, IL-1β, or IL-1β–neutralizing antibody; *n* = 3 independent experiments. (**G**) Serial replating assays of *p53^+/+^* and *p53^R248W/+^* BM cells in the presence or absence of IL-1β; *n* = 3 independent experiments performed in duplicate. (**H** and **I**) MYC targets (**H**) and inflammatory response (**I**) gene signatures were significantly upregulated in IL-1β*–*treated *p53^R248W/+^* LSKs compared with IL-1β*–*treated *p53^+/+^* LSKs; *n* = 3 biological replicates. The comparison among multiple groups was evaluated with 1-way ANOVA (**B**, **D**, and **E**) or 2-way ANOVA (**C**, **F**, and **G**). Statistical significance: *P < 0.05, **P < 0.01, ***P < 0.001, ****P < 0.0001.

To further understand how inflammatory stress confers a competitive advantage to p53 mutant HSPCs, we performed in vitro cell competition assays (Figure 3A). WT competitor cells (CD45.1^+^) show decreased competitiveness after co-culture with p53 mutant cells (CD45.2^+^) for 7 days (Figure 3B). Importantly, WT CD45.1^+^ cells co-cultured with p53 mutant cells show decreased replating potential compared with WT CD45.1^+^ cells co-cultured with WT CD45.2^+^ cells (Figure 3C). However, the number of apoptotic WT CD45.1^+^ cells were comparable between WT and mutant groups (Figure 3D). To understand how p53 mutant cells inhibit the fitness of WT competitor cells in vitro, we measured the levels of cytokines in the condition media utilizing cytokine arrays and observed increased levels of IL-1β, IL-6, and CXCL-9 in mutant cell cultures compared with that of the WT cells (Figure 3E). Notably, WT CD45.1^+^ cells co-cultured with mutant cells showed increased pyroptosis (Figure 3F and Supplemental Figure 3A). Importantly, the enhanced pyroptosis of WT CD45.1^+^ cells seen in the mutant group appeared to be IL-1β dependent, as adding IL-1β to the culture further increased pyroptosis, whereas adding IL-1β–neutralizing antibody brought the level of pyroptosis back to normal (Figure 3F).

IL-1β is known to exert suppressive effects on WT HSPC activity (44, 45). We found that IL-1β treatment decreased the replating potential of WT BM cells, but not that of p53 mutant cells (Figure 3G). To determine the mechanism by which p53 mutant HSPCs are resistant to IL-1β treatment, we performed RNA-seq in *p53^+/+^* and*p53^R248W/+^* LSKs treated with IL-1β (10 ng/mL) or PBS in vitro for 90 minutes (Supplemental Figure 3B). GSEA revealed that HSPC differentiation genes are significantly upregulated in p53 mutant LSKs compared with WT LSKs following IL-1β stimulation (Supplemental Figure 3C). Notably, p53 mutant LSKs treated with IL-1β are enriched with MYC target genes (Figure 3H and Supplemental Figure 3D). IL-1β treatment also increased the expression of genes of the inflammatory response pathway, including IFN-α, IL-6, and IFN-γ (Figure 3I and Supplemental Figure 3E).

### The NLRP1/IL-1β/GSDMD axis is critical for p53 mutant HSPC fitness.

**Figure 4:**
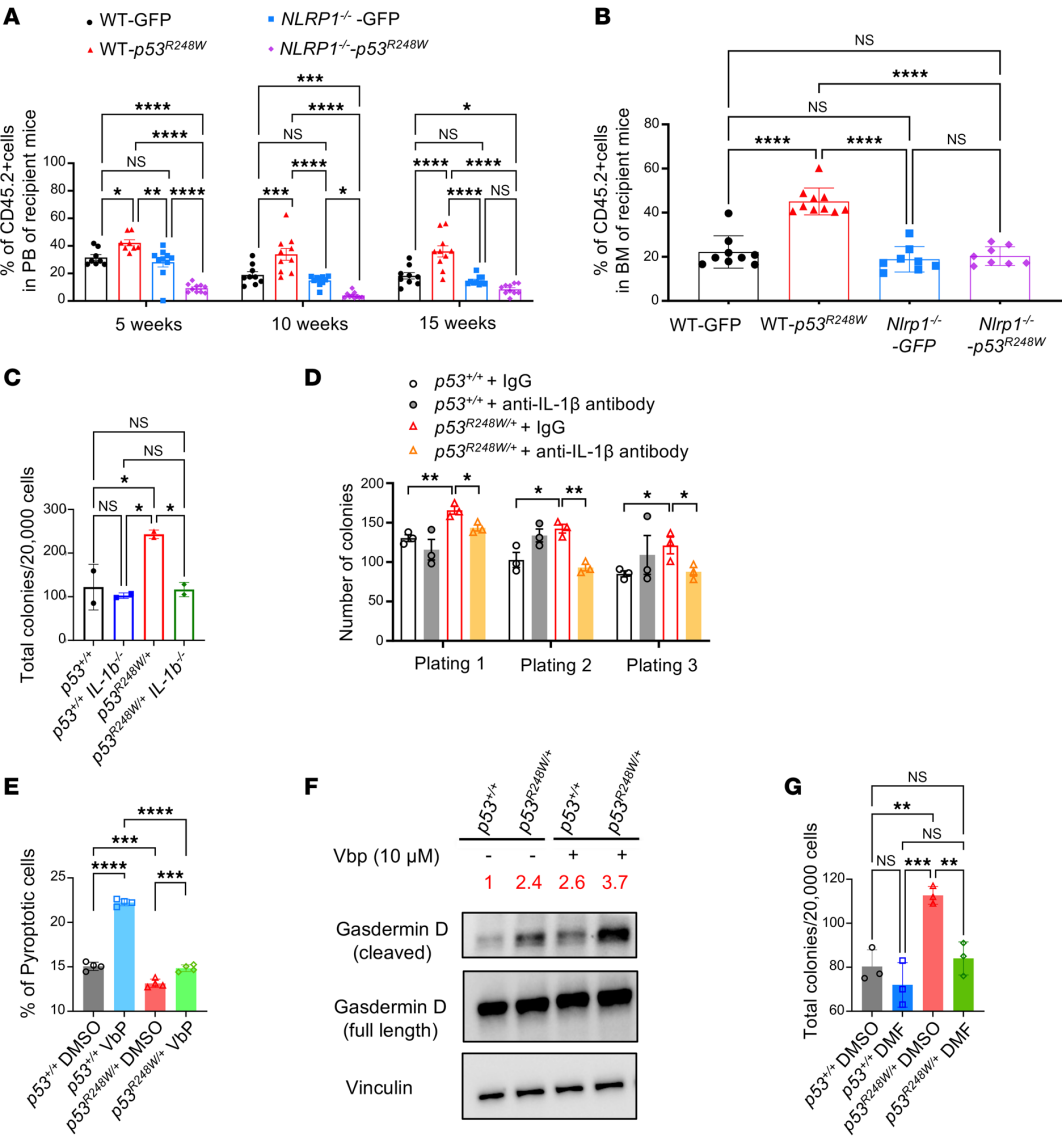
The NLRP1/IL-1β/GSDMD signaling axis is important for p53 mutant HSPC fitness. (**A**) Competitive transplantation experiments using *Nlrp1^+/+^* and *Nlrp1*^−*/*−^ Lin^−^ BM cells expressing GFP or p53^R248W^. The frequency of donor-derived cells (CD45.2^+^) in PB of recipient mice was examined every 5 weeks for 15 weeks; *n* = 7–10 mice per group. (**B**) Loss of *Nlrp1* decreases the engraftment of p53 mutant HSPCs in the BM of recipient mice 15 weeks after transplantation; *n* = 7–10 mice per group. (**C**) Loss of *Il-1b* decreases the colony formation of p53 mutant HSPCs; *n* = 3 biological replicates. (**D**) Serial replating assays of *p53^+/+^* and *p53^R248W/+^* BM cells in the presence of IgG or IL-1β–neutralizing antibody; *n* = 3 independent experiments performed in duplicate. (**E**) *p53^+/+^* and *p53^R248W/+^* BM cells were treated with DMSO or inflammasome activator VbP for 24 h and assayed for pyroptosis; *n* = 3 independent experiments. (**F**) VbP treatment enhances GSDMD maturation in BM cells. (**G**) GSDMD inhibitor dimethyl fumarate (DMF) treatment decreases the colony formation of p53 mutant BM cells; *n* = 3 independent experiments. The comparison among multiple groups was evaluated with 1-way ANOVA (**B**, **C**, **E**, and **G**) or 2-way ANOVA (**A** and **D**). Statistical significance: *P < 0.05, **P < 0.01, ***P < 0.001, ****P < 0.0001.

To determine the role of Nlrp1 in p53 mutant HSPCs, we performed competitive transplantation assays using *Nlrp1^+/+^* and *Nlrp1*^−*/*−^ Lin^−^ cells expressing GFP or mutant p53. We found that loss of *Nlrp1* significantly decreased the repopulating potential of HSPCs expressing mutant p53 in vivo (Figure 4, A and B). To determine the role of Il-1b in p53 mutant HSPCs, we generated *Il-1b*^−*/*−^ *p53^R248W/+^* mice (46). While *Il-1b* deficiency did not affect colony formation of WT HSPCs, loss of *Il-1b* significantly decreased the colony formation of p53 mutant HSPCs (Figure 4C). IL-1β has been shown to be a growth factor for AML cells (47), and we found that IL-1β–neutralizing antibody treatment (48, 49) decreased the replating potential of p53 mutant BM cells compared with p53 mutant cells treated with IgG (Figure 4D).

Given that NLRP1 activation increases IL-1β secretion, inducing pyroptosis of WT HSPCs (43), we treated *p53^+/+^* and *p53^R248W/+^* BM cells with Val-boroPro (VbP), a small molecule activator of inflammasomes (50), and performed pyroptosis analysis. VbP treatment significantly increased pyroptosis of WT BM cells compared with p53 mutant BM cells (Figure 4E). Cytosolic sensing of pathogens assembles inflammasomes, which activate inflammatory caspases to IL-1β and GSDMD. Cleaved GSDMD forms membrane pores, leading to cytokine release and pyroptosis (51, 52). p53 mutant BM cells show increased GSDMD maturation, which is further enhanced by VbP treatment (Figure 4F). While GSDMD inhibitor dimethyl fumarate treatment had no effect on WT BM cells (53), it significantly decreased the colony formation of p53 mutant BM cells (Figure 4G). Collectively, our data suggest that the NLRP1/IL-1β/GSDMD signaling axis is important for the enhanced fitness of p53 mutant hematopoietic cells.

### Heterozygous p53 mutant mice developed MDS with age.

**Figure 5:**
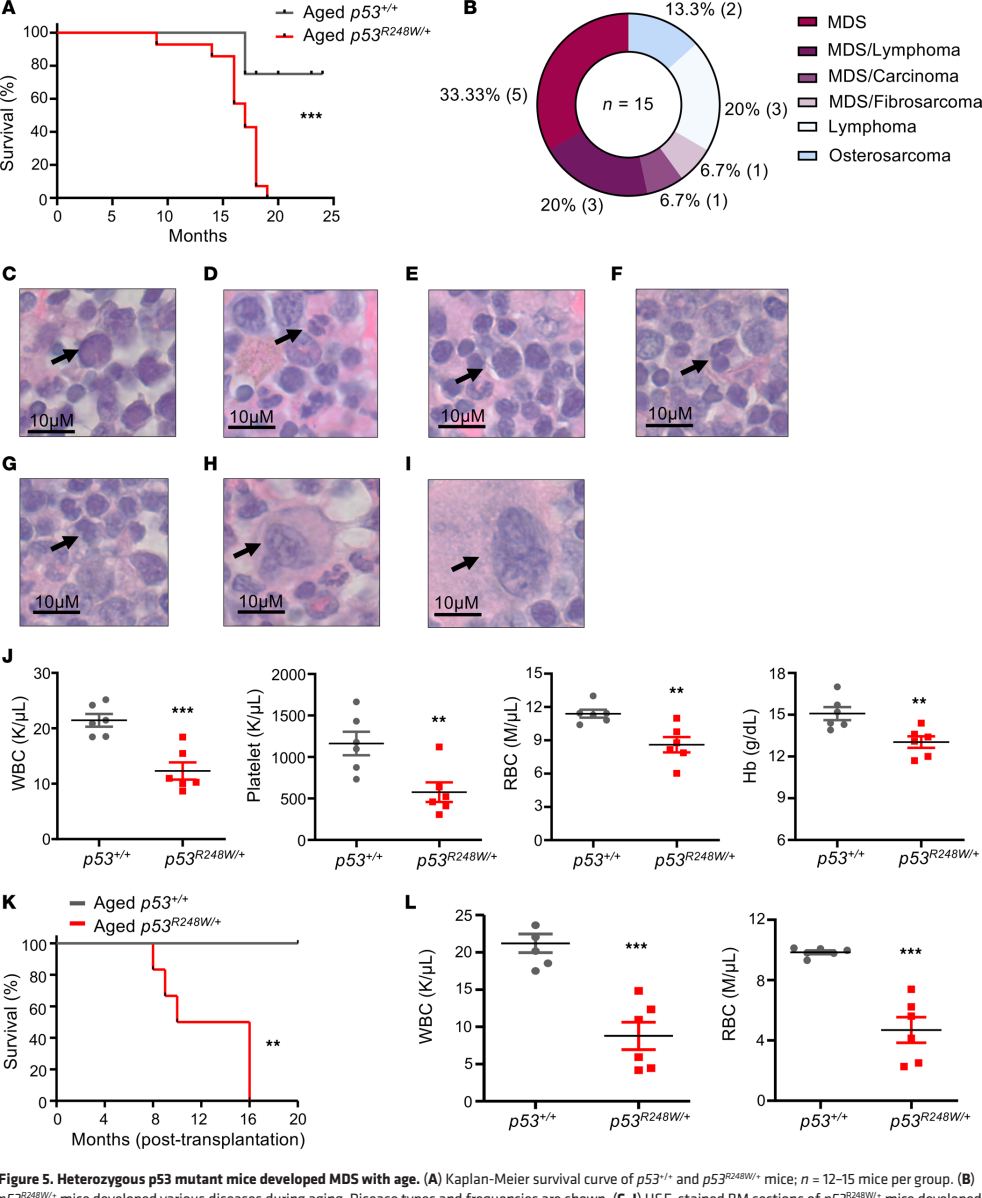
Heterozygous p53 mutant mice developed MDS with age. (**A**) Kaplan-Meier survival curve of *p53*^+/+^ and *p53^R248W/+^* mice; *n* = 12–15 mice per group. (**B**) *p53^R248W/+^* mice developed various diseases during aging. Disease types and frequencies are shown. (**C**–**I**) H&E-stained BM sections of *p53*^R248W/+^ mice developed MDS. Representative images show binucleated myeloid precursor (**C** and **D**), karyorrhexis in erythroid precursors (**E**), erythroid precursors with nuclear budding (**F**), binucleated erythroid precursor (**G**), and a giant megakaryocyte (**H**) compared with a megakaryocyte from age-matched *p53*^+/+^ mice (**I**). Scale bars: 10 μm. (**J**) WBC, platelet, and RBC count as well as hemoglobin levels in PB of *p53^R248W/+^* developed MDS and age-matched *p53*^+/+^ mice; *n* = 6 mice per group. (**K**) Kaplan-Meier survival curve of recipient mice repopulated with BM cells from aged *p53*^+/+^ or *p53^R248W/+^* mice that developed MDS; *n* = 6 mice per group. (**L**) WBC and RBC counts in PB of recipient mice following BM transplantation; *n* = 5 mice per group. The survival curve was determined by the log-rank (Mantel-Cox) test (**A** and **K**). The comparison between 2 groups was evaluated by 2-tailed *t* test (**J** and **L**). Statistical significance: **P < 0.01, ***P < 0.001.

Most homozygous *p53*^−/−^ and *p53^R248W/R248W^* mice develop spontaneous tumors, including lymphoma, thymoma, and sarcoma, and die within 3 to 6 months after birth (54). We aged *p53^+/+^* and heterozygous *p53^R248W/+^* mice for more than 1 year and monitored their survival and tumor development. *p53^R248W/+^* mice showed reduced survival compared with *p53^+/+^* mice and died within 15–18 months after birth (Figure 5A). Approximately 60% of *p53^R248W/+^* mice developed MDS based upon pathological analysis of BM and PB smears, while the remainder of *p53^R248W/+^* mice developed lymphoma and sarcoma (Figure 5B). Aged *p53^R248W/+^* mice showed hypercellular BM compared with age-matched *p53^+/+^* mice (Supplemental Figure 4A). Specifically, aged *p53^R248W/+^* mice showed morphological dysplasia on myeloid precursors, erythroid precursors, and megakaryocytes in the BM (Figure 5, C–I). We also observed morphological dysplasia on *p53^R248W/+^* PB smears, including Pseudo-Pelger-Huët, hypersegmented neutrophils, and nucleated red cells (Supplemental Figure 4B). Significant leukopenia, thrombocytopenia, and anemia were seen in aged *p53^R248W/+^* mice with MDS compared with age-matched *p53^+/+^* mice (Figure 5J). *p53^R248W/+^* mice also show splenomegaly (Supplemental Figure 4, C and D). Moreover, the spleens of aged *p53^R248W/+^* mice have increased frequency of HSPCs (Supplemental Figure 4E). Collectively, the *p53^R248W/+^* mice developed MDS phenotypes, recapitulating some clinical situations seen in MDS patients with *TP53* mutations.

MDS is a clonal disease from mutant MDS stem cells (55, 56). To determine whether mutant p53 driven MDS is transplantable, we injected BM cells from aged *p53^R248W/+^* mice with MDS or age-matched *p53^+/+^* mice into lethally irradiated recipient mice. All recipient mice repopulated with *p53^R248W/+^* cells died within 4 months after transplantation, whereas no mice transplanted with aged *p53^+/+^* BM cells died during this period (Figure 5K). Recipient mice repopulated with *p53^R248W/+^* cells showed decreased WBC and RBC counts compared with recipients of WT BM cells (Figure 5L). Importantly, transplantation of BM cells from *p53^R248W/+^* mice with MDS resulted in lethal MDS and BM failure in all transplant recipients within 4 months (Supplemental Figure 4F), suggesting that the MDS phenotype induced by mutant p53 is transplantable.

### Mutant p53 generates a chronic inflammatory microenvironment during aging.

**Figure 6:**
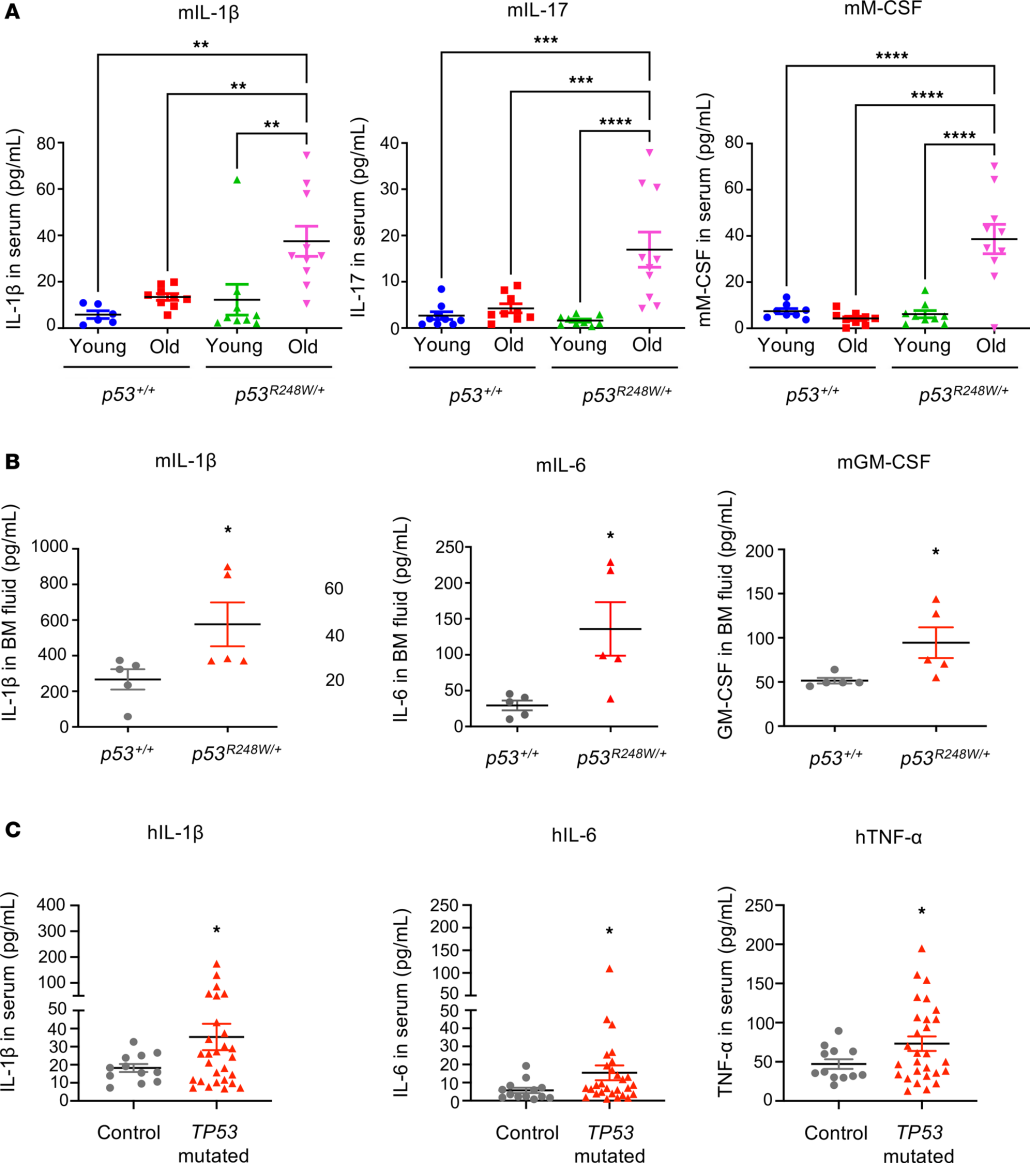
Mutant p53 generates a chronic inflammatory microenvironment during aging. (**A**) Cytokines and chemokines in PB serum of *p53^+/+^* and *p53^R248W/+^* (young and old) mice were assayed using a mouse 31-Plex Cytokine/Chemokine array; *n* = 9 mice per group. (**B**) The levels of cytokines and chemokines in BM fluid of old *p53^+/+^* and *p53^R248W/+^* mice; *n* = 5 mice per group. (**C**) Patients with MDS or AML with *TP53* mutations have increased levels of pro-inflammatory cytokines in their BM compared with the control group (control group, *n* = 12; *TP53*-mutated MDS or AML, *n* = 28). The comparison among multiple groups was evaluated by 2-way ANOVA (**A**). The comparison between 2 groups was evaluated by 2-tailed *t* test (**B** and **C**). Statistical significance: *P < 0.05, **P < 0.01, ***P < 0.001, ****P < 0.0001.

To decipher the mechanism by which mutant p53 drives MDS development, we first measured the levels of cytokines and chemokines in PB serum of young (2–4 months old) *p53^+/+^* and *p53^R248W/+^* mice utilizing cytokine arrays. Cytokine/chemokine levels in young *p53^+/+^* and *p53^R248W/+^* mice were comparable (Figure 6A). Given that chronic and low-grade inflammation develops during aging (9, 10), we then tested whether p53 mutant mice show enhanced systematic inflammation in an age-dependent manner. Notably, we observed increased levels of several pro-inflammatory cytokines, including IL-1β, L-17, and M-CSF, in PB serum of old (15–20 months old) p53 mutant mice compared with that of the age-matched *p53^+/+^* mice (Figure 6A). Given that dysregulated inflammatory signaling within the BM microenvironment has been implicated in MDS development (12), we examined the levels of cytokines and chemokines in BM fluid of old *p53^+/+^* and *p53^R248W/+^* mice and observed increased levels of IL-1β, IL-6, and GM-CSF in the BM of old p53 mutant mice compared with that of age-matched WT mice (Figure 6B).

To further understand the role of chronic inflammation in pathogenesis of *TP53*-mutated myeloid neoplasms, we interrogated cytokine levels in the BM of MDS and AML patients with *TP53* mutations using cytokine arrays. The comparative control group included BMs of lymphoma patients without involvement in disease. Consistent with our observations in p53 mutant mice (Figure 6, A and B), we found increased levels of IL-1β, IL-6, and TNF-α in the BM of MDS or AML patients with *TP53* mutations compared with the control group (Figure 6C). Thus, we demonstrated that mutant p53 generates a chronic inflammatory BM microenvironment during aging, which may contribute to pathogenesis of MDS.

### Murine HSPCs expressing mutant p53 display aberrant pre-mRNA splicing.

**Figure 7:**
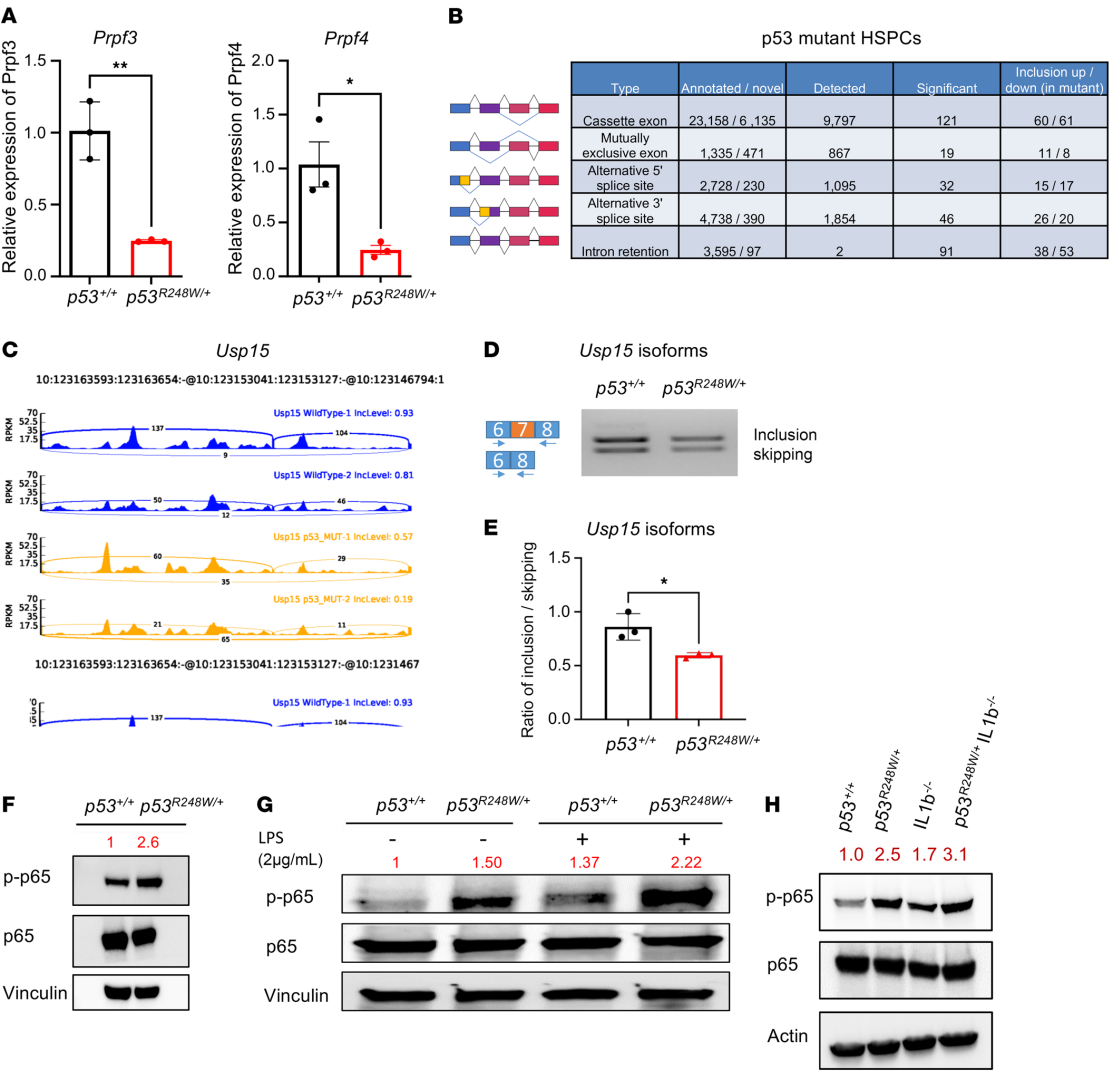
Murine HSPCs expressing mutant p53 display aberrant pre-mRNA splicing. (**A**) Splicing factors *Prpf3* and *Prpf4* were significantly downregulated in middle-aged p53 mutant HSPCs; *n* = 3 biological replicates. (**B**) Table of splicing events altered in middle-aged p53 mutant versus WT HSPCs. (**C**) Sashimi plots across splice junctions surrounding differentially spliced cassette exons in *Usp15*. (**D** and **E**) RT-PCR analysis revealed a significant change in pre-mRNA splicing of *Usp15* in p53 mutant HSPCs; *n* = 3 biological replicates. (**F**) p53 mutant BM cells show enhanced NF-κB activation. (**G**) p53 mutant macrophages show enhanced NF-κB activation following LPS stimulation. (**H**) Loss of *Il-1b* did not alter p65 phosphorylation in p53 mutant BM cells. The comparison between 2 groups was evaluated by 2-tailed *t* test (**A** and **E**). Statistical significance: *P < 0.05, **P < 0.01.

To understand how mutant p53 modulates inflammatory response during aging, we performed RNA-seq studies to compare gene expression in HSPCs isolated from middle-aged (12–14 months old; pre-MDS) *p53^+/+^* and *p53^R248W/+^* mice. Spliceosome genes were significantly downregulated in middle-aged *p53^R248W/+^* HSPCs compared with age-matched *p53^+/+^* HSPCs (Supplemental Figure 4G). We confirmed that the expression of splicing factors, such as *Prpf3* and *Prpf4*, was significantly downregulated in middle-aged (pre-MDS) *p53^R248W/+^* HSPCs (Figure 7A). As *Prpf3* and *Prpf4* play critical roles in spliceosome assembly (57, 58), we speculated that downregulation of *Prpf3* and *Prpf4* in p53 mutant HSPCs may alter pre-mRNA splicing. We conducted alternative splicing analysis utilizing the rMATS algorithm to identify differences in exon inclusion ratio between WT and mutant HSPCs (59, 60). We observed differential splicing of all classes of alternative splicing events, including Cassette Exons, Competing 5′ and 3′ splice sites, Mutually Exclusive Exons, and Retained Introns in *p53^R248W/+^* HSPCs (Figure 7B).

NF-κB is a key mediator of IL-1β/IL-1R and LPS/TLR-4 signaling in immune cells (61). The NF-κB family consists of several proteins, including RelA (commonly known as p65), RelB, c-Rel, p50, and p52, operating as heterodimers or homodimers. Upon IL-1β or LPS stimulation, p65 is phosphorylated and translocated into the nucleus to activate NF-κB target genes (62). Notably, p53 mutant HSPCs show dysregulated pre-mRNA splicing in key regulators of NF-κB such as *Usp15* (Figure 7C). p53 mutant HSPCs mainly express the short isoform of *Usp15* (Figure 7, D and E). Usp15 is a known activator of NF-κB (63), and we found that p53 mutant BM cells have high levels of p65 phosphorylation in steady state (Figure 7F). We also observed that p53 mutant macrophages displayed elevated levels of p65 phosphorylation in steady state compared with WT cells, which was further enhanced by LPS stimulation (Figure 7G). To determine whether the baseline increase of NF-κB in *p53^R248W/+^* BM cells is due to elevated IL-1β systemically or is a cell intrinsic mechanism, we examined p65 phosphorylation in *p53^R248W/+^Il-1b*^−*/*−^ BM cells and found that loss of *Il-1b* did not alter the level of p65 phosphorylation in mutant cells (Figure 7H).

### Human HSPCs and primary MDS cells with TP53 mutations display aberrant pre-mRNA splicing.

**Figure 8:**
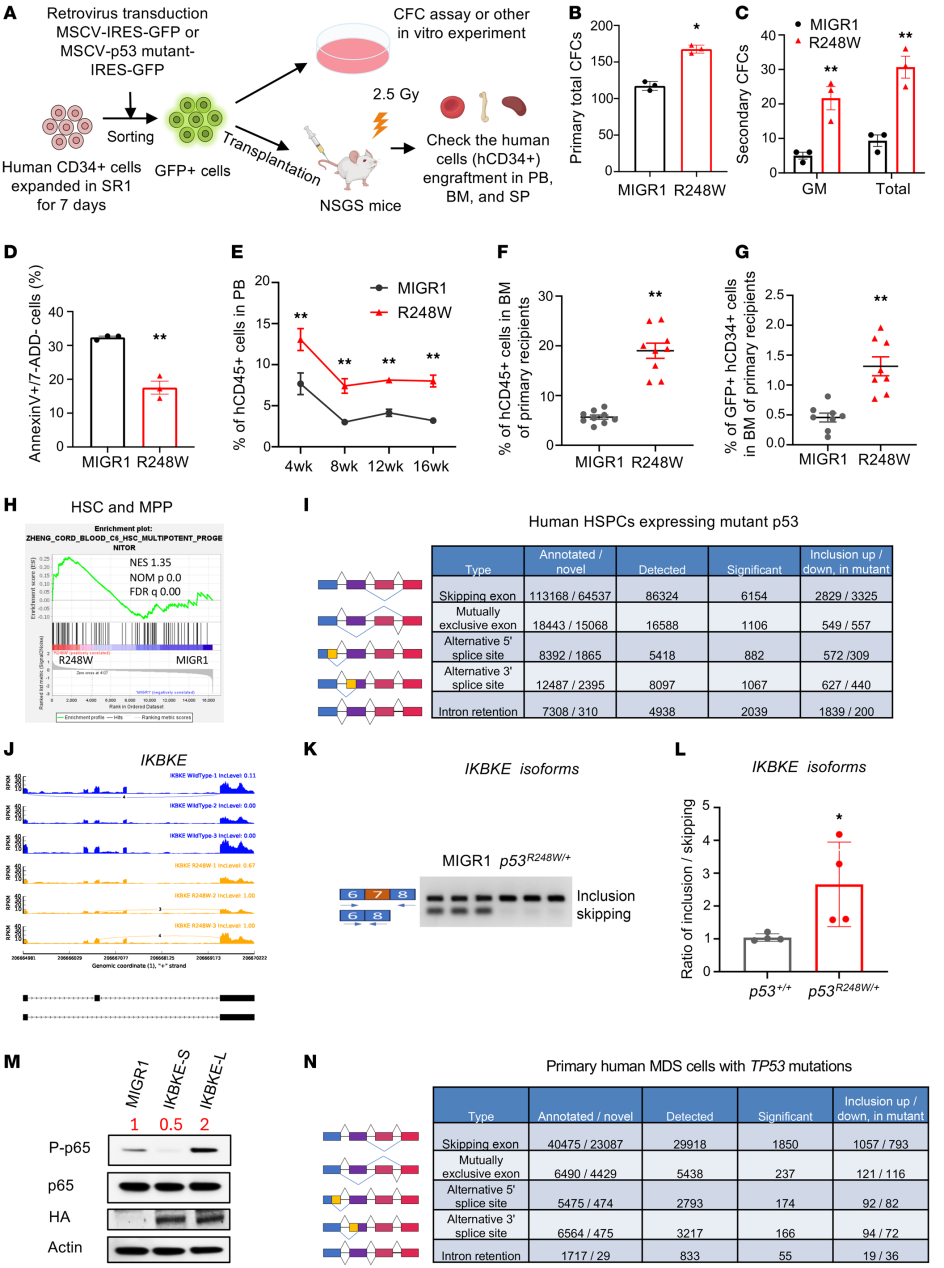
Human HSPCs and primary human MDS cells with *TP53* mutations display dysregulated pre-mRNA splicing. (**A**) Schematic of in vitro and in vivo experiments using human HSPCs expressing mutant p53. CFC, colony-forming cell. (**B** and **C**) Mutant p53 enhances the colony formation of human HSPCs; *n* = 3 biological replicates. (**D**) Human HSPCs expressing mutant p53 show decreased apoptosis following irradiation; *n* = 3 biological replicates. (**E**) Ectopic mutant p53 expression increases the engraftment of human HSPCs in PB of NSGS mice; *n* = 9 mice per group. (**F**) Ectopic mutant p53 expression increases the engraftment of human HSPCs in the BM of NSGS mice at 16 weeks; *n* = 9 mice per group. (**G**) Mutant p53 expands human HSPCs in the BM of NSGS mice at 16 weeks; *n* = 9 mice per group. (**H**) Human HSC and multipotent progenitor cell gene signatures are significantly enriched in human CD34^+^ cells expressing mutant p53. (**I**) Table of splicing events altered in human CD34^+^ cells expressing mutant p53 compared with cells WT for p53; *n* = 3 biological replicates. (**J**) Sashimi plots across splice junctions surrounding differentially spliced cassette exons in *IKBKE*. (**K** and **L**) RT-PCR analysis revealed significant changes in pre-mRNA splicing of *IKBKE* in human HSPCs expressing mutant p53; *n* = 4 biological replicates. (**M**) IKBKE-L, but not IKBKE-S, enhances p65 phosphorylation in MDSL cells. (**N**) Table of splicing events altered in human MDS cells with *TP53* mutations compared with the control group (control, *n* = 2; *TP53* mutated MDS, *n* = 3). The comparison between 2 groups was evaluated by 2-tailed *t* test (**B**–**D**, **F**, **G**, and **L**). The comparison among multiple groups was evaluated by 2-way ANOVA (**E**). Statistical significance: *P < 0.05, **P < 0.01.

To determine the role of mutant p53 in human HSPCs, we introduced GFP or p53^R248W^ into human CD34^+^ cells using retroviruses (Figure 8A) (64). The transduction efficiency was approximately 20%–40% (Supplemental Figure 5A). Mutant p53 maintained CD34 expression in human HSPCs (Supplemental Figure 5, B and C). We sorted transduced CD34^+^ cells (GFP^+^) and performed CFU assays. The ectopic expression of p53^R248W^ enhanced the colony formation of human CD34^+^ cells compared with control viruses (MIGR1) transduced with CD34^+^ cells (Figure 8, B and C). To determine the response of human HSPCs to irradiation, we irradiated human CD34^+^ cells in vitro and performed apoptosis and CFU assays. Human CD34^+^ cells expressing mutant p53 show decreased apoptosis compared with human cells expressing WT p53 (Figure 8D). Mutant p53 also inhibited erythroid differentiation of human HSPCs (Supplemental Figure 5, D and E). To assess the long-term reconstituting ability of human CD34^+^ cells expressing mutant p53, we transplanted human CD34^+^ cells expressing mutant p53 or empty vector (MIGR1) into sublethally irradiated (2.5 Gy) NSGS mice (65) and examined the engraftment of human cells (hCD45^+^) in the PB and BM of recipient mice. Ectopic expression of mutant p53 increased the engraftment of human hematopoietic cells in PB, BM, and spleen of NSGS mice at 16 weeks after transplantation (Figure 8, E–G, and Supplemental Figure 6, A–E). Furthermore, NSGS mice repopulated with p53 mutant HSPCs showed splenomegaly (Supplemental Figure 6, F and G). THP1 is a human monocyte cell line that can differentiate into macrophages following PMA treatment. We introduced MIGR1 or p53^R248W^ into THP1 cells and differentiated them into macrophages. We treated macrophages with LPS for 4 h and examined the levels of full-length NLRP1 and active NLRP1 (NLRP1-NT) in these cells. Consistent with our findings in murine macrophages (Figure 2G and Supplemental Figure 2D), THP1 macrophages expressing mutant p53 had increased levels of full-length NLRP1 at baseline and LPS treatment increased the level of NLRP1-NT in mutant cells (Supplemental Figure 7A). These findings suggest that the impact of mutant p53 on NLRP1 activation in hematopoietic cells is conserved across species.

To determine the mechanism by which mutant p53 enhances the repopulating potential of human HSPCs, we performed RNA-seq studies in human CD34^+^ cells purified from the BM of NSGS mice at 16 weeks following transplantation. GSEA revealed that HSC and multipotent progenitor gene signatures are positively enriched in human HSPCs expressing mutant p53 compared with those of the WT cells (Figure 8H and Supplemental Figure 7B). We then conducted alternative splicing analysis to identify differences in exon inclusion ratio between human CD34^+^ cells expressing GFP and mutant p53 (59, 60). Consistent with our findings in murine HSPCs (Figure 7B), human HSPCs expressing mutant p53 displayed aberrant pre-mRNA splicing events compared with WT HSPCs (Figure 8I). Notably, human HSPCs expressing mutant p53 showed dysregulated pre-mRNA splicing in *IKBKE* (inhibitor of nuclear factor kappa B kinase subunit epsilon) (Figure 8J), a key regulator of NF-κB (66, 67). p53 mutant HSPCs mainly expressed the long isoform of IKBKE (IKBKE-L) (Figure 8, K and L). To determine the impact of IKBKE isoforms on NF-κB activation, we introduced HA-tagged IKBKE-L or IKBKE-S into human MDSL cells using retroviruses and examined NF-κB activation in transduced cells. We found that IKBKE-L, but not IKBKE-S, enhanced p65 phosphorylation in MDSL cells (Figure 8M).

To determine whether mutant p53 alters pre-mRNA splicing in primary human MDS cells, we performed RNA-seq in primary MDS cells with *TP53* mutations and a control group with WT *TP53*. All human samples were free of known splicing factor mutations. Splicing analysis revealed that primary human MDS cells with *TP53* mutations display aberrant pre-mRNA splicing compared with control hematopoietic cells with WT *TP53* (Figure 8N).

## Discussion

*TP53* mutations are present in individuals with CHIP and CCUS (1–5). *TP53* is also frequently mutated in myeloid neoplasms (26, 27). Some individuals with *TP53*-mutant CHIP and CCUS develop hematological malignancies later in their life (3–7). However, how inflammatory stress inhibits WT HSPC fitness and confers a competitive advantage to p53 mutant HSPCs is largely unexplored. We found that inflammatory stress enhances the fitness of p53 mutant HSPCs in transplantation assays. Notably, p53 mutant HSPCs manifest a hyperactive NLRP1/IL-1β signaling pathway, promoting cell proliferation and survival. While WT HSPCs are sensitive to inflammatory stress induced by LPS stimulation, p53 mutant HSPCs are resistant to LPS treatment. Thus, the growth advantage seen in p53 mutant HSPCs is likely due to elevated NLRP1/IL-1β signaling. Our data also suggest that p53 mutant HSPCs benefit from inflammatory signals as the colony formation capability of mutant HSPCs depends on IL-1β.

NLRP3 is activated in HSCs during aging, and aged WT HSCs show increased levels of pyroptosis (68). *Tet2*-deficient macrophages exhibited increased secretion of IL-1β that is mediated by NLRP3, and the clonal expansion of *Tet2*-deficient hematopoietic cells contributes to the exacerbated atherosclerosis in mice (69). Low- to high-risk MDS HSPCs, which usually have WT *TP53*, manifest an activated NLRP3 inflammasome (40). Notably, mutant p53 increases the chromatin accessibility to *NLRP1*, but not *NLRP3*, in HSPCs, leading to NLRP1 activation. Furthermore, loss of NLRP1 significantly decreased the levels of pro-inflammatory cytokines secreted from p53 mutant macrophages. Thus, our findings suggest that mutant p53 activates NLRP1 and NLRP1 is a key mediator of inflammatory response in p53 mutant HSPCs.

IL-1β is important for regulating the immune response in the acute setting; however, it can also promote disease development in the chronic setting (45, 51). We observed increased levels of IL-1β in p53 mutant macrophages following LPS stimulation. In addition, we found that increased pyroptosis seen in WT competitor cells co-cultured with p53 mutant BM cells depend on IL-1β. Thus, IL-1β is a key mediator of mutant p53–driven inflammatory response in hematopoietic cells. While IL-1β treatment inhibits WT HSPC function (45), we found that p53 mutant HSPCs are not sensitive to IL-1β exposure. To understand the underlying mechanisms, we performed RNA-seq studies and found that MYC target gene signatures are enriched in p53 mutant HSPCs following IL-1β treatment. Given that IL-1β treatment is known to decrease WT HSPC proliferation and reduce protein translation and synthesis (45), our findings suggest that altered response to inflammation may promote p53 mutant HSPC survival and provide these HSPCs with a competitive advantage over WT cells. Our GSEA also revealed that HSPC differentiation genes are significantly enriched in p53 mutant LSKs following IL-1β stimulation. Previous studies have shown that the increased expression of differentiation genes induced by Il-1β treatment is reversed by *Cebpa* deficiency, leading to selection for mutant cells (70). Thus, it appears that *CEBPA* and *TP53* mutation can overcome Il-1b–mediated suppression of HSPC fitness through distinct but overlapping mechanisms.

A growing body of evidence, primarily from studies involving *Tet2-* and *Dnmt3a*-KO mice and stem cell transplantation models of CHIP (15, 16), suggests that dysregulated inflammation may contribute to mutant HSPC expansion. For example, we and others found that inflammatory stress promotes the clonal expansion of *Tet2* mutant HSPCs (15, 36). TET2 appears to restrain inflammatory gene expression in macrophages, manifested by increased expression of LPS-induced genes in *Tet2*^−*/*−^ macrophages (36). Recently, IFN-γ signaling pathway has been implicated in promoting *Dnmt3a*-null HSPC expansion (16). Resistance to inflammation appears to underly enhanced fitness seen in *Asxl1-*deficient HSPCs (17). These studies suggest that the mechanisms by which inflammatory stress drives CHIP emergence and progression may be mutation specific.

We utilized a lethal dose of irradiation to generate chimeric mice for competition studies (24). However, irradiation is known to promote tissue damage and inflammation (11, 12). This inflammation could account for the selection for p53 mutant HSPC even in the absence of pI:pC or other additional inflammatory stimuli. To alleviate the impact of radiation on the host inflammatory state, we will utilize less inflammatory conditioning methods such as low-dose busulfan or nonmyeloablative whole BM transplants to establish mouse models of CHIP in the future.

There have been no studies on the functional impact of *TP53* mutations on MDS pathogenesis (26, 27), so it is important to develop mouse models of myeloid neoplasms with *TP53* mutations. We reported that mutant p53 drives MDS development in mice during aging and that mutant p53–driven MDS is transplantable. *p53^R248W/+^* mice developed diverse disease phenotypes after a long latency, suggesting that additional cooperating mutations may contribute to MDS development. It is possible that *TP53* mutations may cooperate with *DNMT3A*, *TET2*, *ASXL1*, or other mutations to drive disease development (27). The majority of *TP53*-mutated MDS patients have multiple-hit *TP53* mutations, and this state predicted risk of death and leukemic transformation (27). Thus, *TP53* allelic state may be critical for prognosis and treatment response. Given that *p53^R248W/+^* mice are global knockin mice, and these mice also develop lymphoma and other tumors (24, 25, 54), conditional knockin mice of hotspot *TP53* mutations will be needed to determine the impact of *TP53* allelic state in MDS pathogenesis and develop preclinical models for testing MDS therapeutics.

During aging, chronic and low-grade inflammation develops (9, 10), and we observed increased levels of IL-1β and IL-6 in the BM of old p53 mutant mice and in patients with MDS or AML with *TP53* mutations. Thus, mutant p53 generates a chronic inflammatory BM microenvironment during aging, which may contribute to pathogenesis of MDS. Li-Fraumeni syndrome (LFS) predisposes individuals to hereditary cancer linked to *TP53* germline mutations (71). Many tumor types are found in LFS patients, including sarcomas, brain tumors, breast cancer, and hematological malignancies (71). It will be interesting to examine cytokine and chemokine levels in LFS patients and their healthy, mutation-negative relatives to better understand the role of *TP53* mutations in cancer development in patients with LFS.

To determine the mechanism by which mutant p53 drives MDS development during aging, we performed RNA-seq studies and found that both murine and human HSPCs expressing mutant p53 exhibit aberrant pre-mRNA splicing. Notably, we found that primary human MDS cells with *TP53* mutations show dysregulated pre-mRNA splicing, and the extent of changes in alternative splicing in primary human MDS cells with *TP53* mutations is similar to what is seen in MDS patients with spliceosome mutations (72, 73), underscoring the important role that mutant p53 plays in regulating pre-mRNA splicing.

While chronic inflammation has been implicated in the clonal evolution of *TP53*-mutated therapy-related myeloproliferative neoplasms (74), how mutant p53 regulates inflammatory response in HSPCs remains elusive. We found that mutant p53 alters pre-mRNA splicing in key regulators of NF-κB, such as Usp15 and IKBKE, leading to enhanced NF-κB activation in hematopoietic cells, thereby driving MDS development. Altered RNA splicing by mutant p53 has been shown to activate oncogenic RAS signaling in pancreatic cancer (75); thus, our findings suggest that mutant p53 may dysregulate RNA splicing in a context-dependent manner to promote tumorigenesis.

Given that CHIP has become an emerging public health issue that affects 15%–20% of individuals age 70 or above (1–7), preventing the progression of CHIP to life-threatening diseases such as MDS and AML will have huge health benefits. We found that loss of NLRP1 decreases the engraftment of p53 mutant HSPCs in transplantation assays. As there is no NLRP1-specific inhibitor available (41, 42), the impact of pharmacological inhibition of NLRP1 on p53 mutant HSPCs awaits future investigation. We predict that p53 mutant HSPCs will be sensitive to NLRP1-specific inhibitor treatment both in vitro and in vivo. IL-1β has been shown to expand *CEBPα*-deficient hematopoietic progenitors in vivo (70). We found that IL-1β–neutralizing antibody treatment decreases the colony formation of p53 mutant HSPCs. Furthermore, p53 mutant HSPCs are sensitive to GSDMD inhibitor in vitro. IL-1β–neutralizing antibody (canakinumab) is in different phases of clinical trials (76) and several GSDMD inhibitors are FDA-approved drugs (77), so testing these drugs in individuals with *TP53*-mutant CHIP may be of clinical benefit.

Recently, *TP53*-mediated CH has been shown to confer increased risk for incident atherosclerotic disease (78). However, loss of p53 did not alter the expression of IL-1β and IL-6 in macrophages (78), suggesting that *TP53* mutations may utilize distinct mechanisms to drive the development of different age-associated diseases.

## Methods

### Sex as a biological variable.

Our study examined male and female animals, and similar findings are reported for both sexes.

### Animals.

*p53^R248W/+^* mice used in our studies have been described previously (20, 24, 25, 54). WT C57BL/6 (CD45.2^+^), Boy/J (CD45.1^+^), and F1 mice (CD45.2^+^CD45.1^+^) mice were obtained from an onsite core breeding colony. *Nlrp1*^−/−^ mice were generated in-house (43). *Il-1b*–KO (46) and NSGS (65) mice were obtained from The Jackson Laboratory.

### Flow cytometry.

Flow cytometry analysis of murine and human HSPCs in PB, BM, and spleen of recipient mice was performed as described previously (20, 24, 25, 64). Flow cytometry antibodies are listed in Supplemental Dataset 1.

### Determination of the impact of inflammatory stress on HSPCs in vivo.

To determine whether pI:pC treatment expands p53 mutant HSPCs in vivo, we performed competitive BM transplantation assays. To mimic CH, the ratio of donor cells/competitor cells was 1:10. We transplanted 1 × 10^5^ *p53^+/+^* and *p53^R248W/+^* BM cells (CD45.2^+^) together with 1 × 10^6^ competitor BM cells (CD45.1^+^) into lethally irradiated F1 mice (CD45.1^+^CD45.2^+^). Eight weeks following transplantation, recipient mice were treated with PBS or pI:pC (25 μg/kg, i.p.) twice a week for 4 weeks (34). The frequency of donor-derived cells (CD45.2^+^) in PB was assayed by flow cytometry analysis.

To determine whether LPS treatment expands p53 mutant HSPCs in vivo, we transplanted 1 × 10^5^ *p53^+/+^* and *p53^R248W/+^* BM cells (CD45.2^+^) together with 1 × 10^6^ competitor BM cells (CD45.1^+^) into lethally irradiated F1 mice (CD45.1^+^CD45.2^+^). Four weeks following transplantation, recipient mice were treated with PBS or LPS (1 mg/kg, i.p.) every other day for 4 weeks (35). The frequency of donor-derived cells (CD45.2^+^) in PB was assayed by flow cytometry analysis every 4 weeks for 16 weeks.

To determine the impact of LPS treatment on HSPC function, we treated *p53^+/+^* and *p53^R248W/+^* mice with PBS or LPS (1 mg/kg, i.p.) every other day for 4 weeks. We then transplanted 5 × 10^5^ BM cells from PBS or LPS-treated *p53^+/+^* and *p53^R248W/+^* mice (CD45.2^+^) plus 5 × 10^5^ competitor BM cells (CD45.1^+^) into lethally irradiated F1 mice (CD45.1^+^CD45.2^+^). The frequency of donor-derived cells (CD45.2^+^) in PB was assayed by flow cytometry analysis every 4 weeks for 16 weeks. At 16 weeks following primary transplantation, we transplanted 3 × 10^6^ BM cells isolated from primary recipient mice into lethally irradiated secondary recipient mice. The frequency of donor-derived cells (CD45.2^+^) in PB and BM was assayed by flow cytometry analysis.

### Stem and progenitor cell assays.

The CFU assays using murine or human HSPCs were performed as described previously (20, 24, 25, 64).

### Generation of BM-derived macrophages.

We cultured low-density BM mononuclear cells from *p53^+/+^* and *p53^R248W/+^* mice in IMDM supplemented with 20% FBS and 100 ng/mL GM-CSF (Pepro Tech) for 7 days (36). 5 × 10^6^ BM-derived macrophages were starved overnight in the absence of growth factors. Macrophages were treated with LPS (100 ng/mL) for 12 h (36). Supernatants were collected and subjected to cytokine array analysis (Eve Technologies, Mouse 31-Plex Cytokine/Chemokine Array). Cytokine array data are provided in Supplemental Dataset 2.

### Generation of retroviruses and infection of murine HSPCs.

Retroviruses were produced by transfection of Phoenix E cells with the MIGR1 control (MSCV-IRES-GFP) or MIGR1 full-length mutant p53 cDNA plasmid (MSCV-p53^R248W^-IRES-GFP) according to standard protocols (20, 24, 25). Lineage-negative cells isolated from WT and *Nlrp1*^−*/*−^ mice were infected with high-titer retroviral suspensions in the presence of retronectin (Takara). Forty-eight hours after infection, the transduced cells (GFP^+^) were sorted by FACS.

### In vitro cell competition assays.

We cultured WT or mutant BM cells (CD45.2^+^) with competitor BM cells (CD45.1^+^) from B6.SJL mice. We generated 3 groups of BM cells in vitro: (a) 1 × 10^6^ WT CD45.1^+^ cells; (b) 5 × 10^5^ WT CD45.1^+^ + 5 × 10^5^ WT CD45.2^+^ cells; and (c) 5 × 10^5^ WT CD45.1^+^ + 5 × 10^5^ mutant CD45.2^+^ cells. We cultured 3 groups of cells in serum-free medium supplemented with hematopoietic cytokines, including FLT3 ligand, TPO, SCF, IL-3, and IL-6, for 7 days. We then sorted an equal number of live CD45.1^+^ cells from each group and performed proliferation, colony formation, apoptosis, and pyroptosis assays. In addition, we measured the levels of cytokines in conditioned medium utilizing cytokine arrays. Cytokine array data are provided in Supplemental Dataset 2.

### RNA- and ATAC-seq assays.

RNA-seq assays using murine HSPCs and data analysis were performed as described previously (24). We analyzed changes in chromatin accessibility in *p53^+/+^* and *p53^R248W/+^* LSKs by performing ATAC-seq (37). To ensure reproducibility, 2 biological replicates were performed, and 5 × 10^4^ LSK cells were pooled from age- and sex-matched littermates. Quality control of Fastq files was done by the FastQC tool. TopHat2 was used to align sequences on the mm9 genome. Reads from BAM files were filtered according to mapping quality value (mapq value, option -q 10). Then, peaks were detected on these BAM files by macs2. BigWig files were generated from bedgraph files (37). RNA- and ATAC-seq data are available in the Gene Expression Omnibus (GEO) under accession number GSE175370.

### RNA splicing analysis.

Alternative splicing events were identified by replicate Multivariate Analysis of Transcript Splicing (rMATS, version 4.1.0) (60). We adopted the Gencode annotation (GRCh38.p13, hg38) to define the splicing events (79). For each splicing event, the proportions of inclusion (*I*, the event occurred) and exclusion (*S*, otherwise) were quantified based on the junction reads supporting the respective isoforms in the RNA-seq alignment files. Changes in the inclusion level (*L_I_*) across the control and mutant samples, as well as the statistical significance, were evaluated; this computational analysis was performed in murine HSPCs, human HSPCs, and primary human MDS cells. Differential alternative splicing events were selected under FDR < 0.05. In addition, differential splicing events that further satisfied (a) having ≥ 10 support junction reads in ≥ 60% of samples in the control and mutant group and (b) absolute fold change of *L_I_* > 10% between control and mutant were highlighted.

See Supplemental Methods for additional information.

### Statistics.

Statistical analyses were performed with GraphPad Prism 9 software (GraphPad Software Inc). All data are presented as mean ± SEM. The sample size for each experiment and the replicate number of experiments are included in the figure legends. Statistical analyses were performed using 2-tailed *t* test where applicable for comparison between 2 groups, and a 1- or 2-way ANOVA test was used for experiments involving more than 2 groups. Representative Western blot and PCR results are shown from at least 3 biologically independent experiments. Representative flow cytometry profiles are shown from 3 biological replicates. Statistical significance was defined as *P* < 0.05, *P* < 0.01, *P* < 0.001, and *P* < 0.0001.

### Study approval.

MDS, AML, and control samples were collected at Northwestern University Memorial Hospital after informed consent. Protocols for sample handling and data analysis were approved by the Northwestern University Ethics Committee. All experiments involving primary human patient samples were performed in compliance with the Declaration of Helsinki. Furthermore, all animal experiments have received ethical approval from both Indiana and Northwestern University IACUCs. All mice were maintained in Indiana and Northwestern University animal facilities according to IACUC-approved protocols.

### Data availability.

Data are available in GEO (accession number GSE175370), in the Supporting Data Values file, or from the corresponding author upon request.

## Author contributions

SC, SB, S Vemula, and YY: conceptualization, data curation, formal analysis, methodology, writing (original draft), writing (review), and editing. ES, HG, R Li, and FB: investigation, data curation, methodology, and formal analysis. SCN, PJ, and GES: investigation, data curation, and formal analysis. DAS, HC, WC, SX, R Luo, MAA, MLC, JPR, AF, SZ, TMM, M Sotelo, HP, SKH, S Veranga, MA, M Shumilina, KR, YJ, HL, and IK: investigation. RK, YA, JKA, EAE, LAG, CRZ, SLM, BAC, HSB, ZG, LDM, SAS, SH, YD, LCP, and SLM: resources. M Sukhanova, Yunlong Liu, and OAW: conceptualization, resources, formal analysis, supervision, investigation, visualization, writing (original draft), writing (review), and editing. Yan Liu: conceptualization, resources, formal analysis, investigation, visualization, supervision, funding acquisition, project administration, writing (original draft), writing (review), and editing.

## Funding support

This work is the result of NIH funding, in whole or in part, and is subject to the NIH Public Access Policy. Through acceptance of this federal funding, the NIH has been given a right to make the work publicly available in PubMed Central.

- National Institute of Diabetes and Digestive and Kidney Diseases Cooperative Center of Excellence in Hematology grant U54 DK106846 (to the Flow Cytometry Core and In Vivo Therapeutic Core Laboratories at the Indiana University School of Medicine).
- NIH grants R01 CA298152, R01 HL150624, R56 DK119524, and R56 AG052501 (to Yan Liu).
- Department of Defense awards W81XWH-18-1-0265 and DoD W81XWH-19-1-0575 (to Yan Liu).
- The Leukemia & Lymphoma Society (LLS) Translational Research Program award 6581-20 (to Yan Liu).
- St. Baldrick’s Foundation Scholar award (to Yan Liu).
- LLS Career Development Special Fellow award (to SC).
- American Society of Hematology Research Restart award (to SC).
- Lady Tata Memorial International Awards for Research in Leukaemia (to SC).
- NIH F31 award F31HL160120 (to SB).
- NIH F32 award 1F32CA203049-01 (to SCN).

## Supplementary Material

Supplemental data

Supplemental data set 1

Supplemental data set 2

Unedited blot and gel images

Supporting data values

## Version 1 12/30/2025

In-Press Preview

## Version 2 02/02/2026

Electronic publication
