# Supplementary material for: Mutant p53 promotes clonal hematopoiesis by generating a chronic inflammatory microenvironment
Source: J Clin Invest. 2025 Dec 30;136(3):e184285. doi: 10.1172/JCI184285 (PMC12867160; doi:10.1172/JCI184285)
Supplement: Supplemental data [file jci-136-184285-s329.pdf]

Supplementary information

Mutant p53 promotes clonal hematopoiesis by generating a chronic inflammatory microenvironment

Supplemental methods

Apoptosis and pyroptosis assays. Hematopoietic cells were stained with an anti-Annexin-V antibody and DNA dye DAPI. Apoptosis was assessed by flow cytometry analysis (20, 24-25). For pyroptosis assays, hematopoietic cells were first incubated with binding buffer and then stained with FAM FLICA (ImmunoChemistry) and 7-AAD. Early-stage pyroptotic cells are defined as $FLICA^{+}7\text{-}AAD^{-}$ and late-stage pyroptotic cells are defined as $FLICA^{+}7\text{-}AAD^{+}$ (50).

Isolating bone marrow fluid. Tibias and femurs were harvested from *p53*$^{+/+}$ and *p53*$^{R248W/+}$ mice. All bones were flushed with 250 µL of PBS on ice and centrifuged at 2,000 rpm for 3 minutes at 4°C to remove cells. The bone marrow fluid was then collected and stored at −80°C. To measure the levels of cytokines and chemokines present in the bone marrow fluid, cytokine array was performed (Eve Technologies: Mouse Cytokine / Chemokine Array 31-Plex). Cytokine array data were included as an Excel file in the Supplemental Information.

Culture and transduction of human HSPCs with retroviruses. Human umbilical cord blood $CD34^{+}$ cells were expanded in StemSpan (StemCell Technology) media supplemented with TPO, SCF, FLT3 ligand, and 0.75µM or 1µM SR1 for 7 days (64). Expanded human $CD34^{+}$ cells were infected with high-titer retroviral suspensions in the presence of Retronectin (Takara, Japan).

Forty-eight hours after infection, the transduced cells (GFP^+) were sorted by FACS. GFP^+ cells were used for *in vitro* and *in vivo* hematopoietic stem and progenitor cell assays. We transplanted 3 x 10^5 GFP^+ cells into sub-lethally irradiated (2.5Gy) NSGS mice via tail vein injection. The engraftment of human cells ($hCD45^+$) in PB was determined by flow cytometry every 4 weeks for 16 weeks. At 16 weeks post-transplantation, NSGS mice were sacrificed and the frequency of human cells in bone marrow and spleen were examined.

Quantitative Real-Time PCR. Quantitative Real-Time RT-PCR assays were performed as described previously (24).

Immunofluorescence labeling. $p53^{+/+}$ and $p53^{R248W/+}$ Lin^- BM cells were cultured in a six-well plate with RPMI medium supplemented with cytokines for 24 h followed by incubation for additional 12 h in the absence or presence of pI:pC. The cells were then spun onto slides with a Shandon Cytospin centrifuge. Cells were immediately fixed with 4% formaldehyde for 20 min at room temperature, and then permeabilized with phosphate-buffered saline (PBS) containing 0.2% Triton X-100 at 37ºC for 10 min. After permeabilization fixed cells were washed with PBS containing 0.02% Triton X-100 and then blocked with 3% bovine serum albumin (BSA) for 1hr at room temperature. Cells were then treated with anti-rabbit Phospho-Histone H2A.X (Ser139) (clone 20E3, 9718, Cell Signal) antibodies with 1:350 dilution at 37 ºC for 1 h followed by washing with PBS containing 0.02% Triton X-100. The primary antibodies were detected by treating cells for 1 h at 37 ºC with the appropriate secondary antibodies conjugated to Alexa Fluor-488 (Molecular Probes), at a dilution of 1:250. After incubation, cells were washed again with PBS containing 0.02% Triton X-100, counterstained with 1 mg/mL DAPI (blue) and mounted on microscopic slide with prolong Gold and imaged under microscopy.

Image acquisition and analysis. We performed imaging using the confocal super resolution spinning disk microscopy at the Center for Advanced Microscopy/Nikon Imaging Center (Northwestern University, Chicago, IL). For image acquisition, three-dimensional stacks were obtained through the cell using a Nikon confocal inverted microscope equipped with a Yokogawa CSU-W1 SoRa (super resolution) system and X60 1.4 numerical aperture Plan-Apochromatic differential interface contrast oil immersion objective (Nikon). Images were acquired at room temperature as Z-stacks at 0.25 μm intervals controlled by NIS elements software (Nikon). Images were processed in Fiji ImageJ software and represent maximum-intensity projections of the required z-stacks.

Pathological analysis. We evaluated bone marrow and peripheral blood smears (PBS) to identify dysplasia.

Key features of dysplasia in the BM include: Hypercellularity, increase presence of myelocytes and metamyelocytes (doughnut hole formation in nuclei), bands nuclei (horseshoe shape), and collapsed vascular spaces.

Key features of dysplasia in PB include: Hypersegmentation of neutrophils (more than 4 nuclear segments in the neutrophils), Pseudo-Pelger-Huet, Holly-Jolly body (Nucleated RBC), Polychromatophilic RBC, and Tear drop RBC).

PMA-induced THP-1 cell differentiation. THP-1 cells were differentiated into macrophages using phorbol 12-myristate 13-acetate (PMA). The cells were treated with 200 nM of PMA for 24 hours and then allowed to recover for an additional 24 hours before being used in experiments. Forty-eight hours after the PMA treatment, the cells were stimulated with 1 μg of LPS or PBS for 12 hours and then harvested for Western blot analysis.

Transducing MDSL cells with retroviruses. MIGRI, HA-IKBKE-short isoform, or HA-IKBKE-long isoform were introduced into MDSL cells through retroviral-mediated transduction. 48 hours after transduction, transduced cells (GFP^+) were purified using FACS.

Supplemental Figure Legends

Supplemental Figure 1. (A) Immunofluorescence labeling of Lin^- cells treated with pI:pC at different concentrations. Wildtype p53 (left 3 panels) and mutant p53 (right 3 panels) cells were cytospun on cover slip and immunolabeled for γH2AX (green) after 12 h of pI:pC treatment. Chromosomes were counterstained using DAPI (blue). Bars, 10 µm. (**B**) Quantitation of immunofluorescence experiment shown in supplemental Figure 1A. (**C**) Percentage of donor-derived cells ($CD45.2^+$) in the BM of primary recipient mice; n=10-15 mice per group. (**D**) The frequency of donor-derived cells in the BM of secondary recipient mice; n=9 mice per group. (**E**) $p53^{+/+}$ and $p53^{R248W/+}$ macrophages were stimulated with LPS, and the levels of secreted cytokines and chemokines were assayed using a mouse 31-Plex Cytokine/Chemokine array; n=4 biological replicates. The comparison among multiple groups was evaluated with one-way ANOVA (**B** and **E**) or two-way ANOVA (**C** and **D**). Statistical significance ns: *, $P<0.05$; ***, $P<0.001$; ****, $P<0.0001$, NS, not significant.

Supplemental Figure 2. (**A**) Enriched biological processes of genes associated with significantly upregulated ATAC-seq peaks in $p53^{+/+}$ LSKs analyzed by DAVID Functional Annotation Tool. (**B**) *Cxcl9* acquired significant open chromatin peaks in $p53^{R248W/+}$ LSKs. (**C**) *Cxcl9* is upregulated in $p53^{R248W/+}$ LSKs compared to $p53^{+/+}$ LSKs; n=3 biological replicates. *, $P<0.05$. (**D**) The levels

of Gsdmd, Gsdmd-NT, cleaved Caspase-1, and cleaved Caspase-3 in BM-derived macrophages following LPS treatment were determined by immunoblotting. (**E**) The levels of cGas and Sting in lineage-negative BM cells following pI:pC treatment were determined by immunoblotting. The comparison between two groups was evaluated by two-tailed t-test (**C**). Statistical significance ns: *, P<0.05.

Supplemental Figure 3. (A) Gating strategy for assessing pyroptosis in cell competition assays. (**B**) Schematic of RNA-seq of $p53^{+/+}$ and $p53^{R248W/+}$ LSKs treated with PBS or IL-1β; n=3 biological replicates. (**C-E**) Gene Set Enrichment Analysis (GSEA) revealed that hematopoietic stem and progenitor cell differentiation (C), MYC targets (D), and inflammatory response (E) gene signatures were significantly upregulated in IL-1β-treated $p53^{R248W/+}$ LSKs compared to IL-1β-treated $p53^{+/+}$ LSKs. n=3 biological replicates.

Supplemental Figure 4. (**A**) H&E staining of bone marrow sections from aged $p53^{+/+}$ and $p53^{R248W/+}$ mice. (**B**) Giemsa-stained peripheral blood smears from $p53^{\mathrm{R248W/+}}$ MDS mice. Representative images show Pseudo-Pelger-Huet (a), Hypersegmented neutrophil (b), Howell-Jolly body in RBCs (c), polychromatophilic RBC (d), and tear-drop RBCs (e). (**C**) Representative images of spleens from aged $p53^{+/+}$ and $p53^{R248W/+}$ mice. (**D**) Spleen weight of aged $p53^{+/+}$ and $p53^{R248W/+}$ mice. n=6 mice per group. (**E**) The frequency of HSPCs in the spleen of aged $p53^{+/+}$ and $p53^{R248W/+}$ mice. n=6 mice per group. (**F**) Recipient mice developed MDS and BM failure following transplantation of BM cells from $p53^{R248W/+}$ mice with MDS. Disease types and frequencies are shown. n=6 mice per group. (**G**) Spliceosome genes were significantly downregulated in middle-aged (Pre-MDS) p53 mutant HSPCs compared to age-matched wild type

HSPCs. The comparison between two groups was evaluated by two-tailed t-test (**D**). The comparison among multiple groups was evaluated with one-way ANOVA (**E**). Statistical significance ns: *, P<0.05, **, P<0.01, ***, P<0.001.

Supplemental Figure 5. (**A**) Flow cytometry analysis showing the transduction of human $CD34^+$ cells with retroviruses expressing GFP (MIGR1) or mutant p53. (**B-C**) Mutant p53 increases CD34 expression in human HSPCs. n=3 biological replicates. (**D-E**) Erythroid differentiation of human $CD34^+$ cells expressing GFP or mutant p53 as determined by flow cytometry analysis. n=3 biological replicates. The comparison between two groups was evaluated by two-tailed t-test (**C** and **E**). Statistical significance ns: *, P<0.05, **, P<0.01.

Supplemental Figure 6. (**A**) Flow cytometry analysis of human hematopoietic cells ($hCD45^+$) in peripheral blood of NSGS mice following transplantation. (**B**) Ectopic mutant p53 expression increases the engraftment of human HSPCs in PB of NSGS mice. n= 9 mice per group. (**C**) Ectopic mutant p53 expression increases the engraftment of human HSPCs in the BM of NSGS mice at 16 weeks. n= 9 mice per group. (**D-E**) Ectopic mutant p53 expression increases the engraftment of human HSPCs in the spleen of NSGS mice at 16 weeks. n= 9 mice per group. (**F-G**) NSGS mice transplanted with human $CD34^+$ cells expressing mutant p53 show splenomegaly. n=9 mice per group. The comparison between two groups was evaluated by two-tailed t-test (**C**, **D, E** and **G**). The comparison among multiple groups was evaluated with two-way ANOVA (**B**). Statistical significance ns: **, P<0.01.

Supplemental Figure 7. (A) Differentiation of THP-1 cells were induced by PMA. THP-1 macrophages were stimulated with 1 μg of LPS for 4 hours and the levels of NLRP1 and NLRP1-NT were determined by immunoblotting. (**B**) Human $CD34^+$ multipotent progenitor cell gene signatures are significantly enriched in human $CD34^+$ cells expressing mutant p53.

Supplemental Figure 1

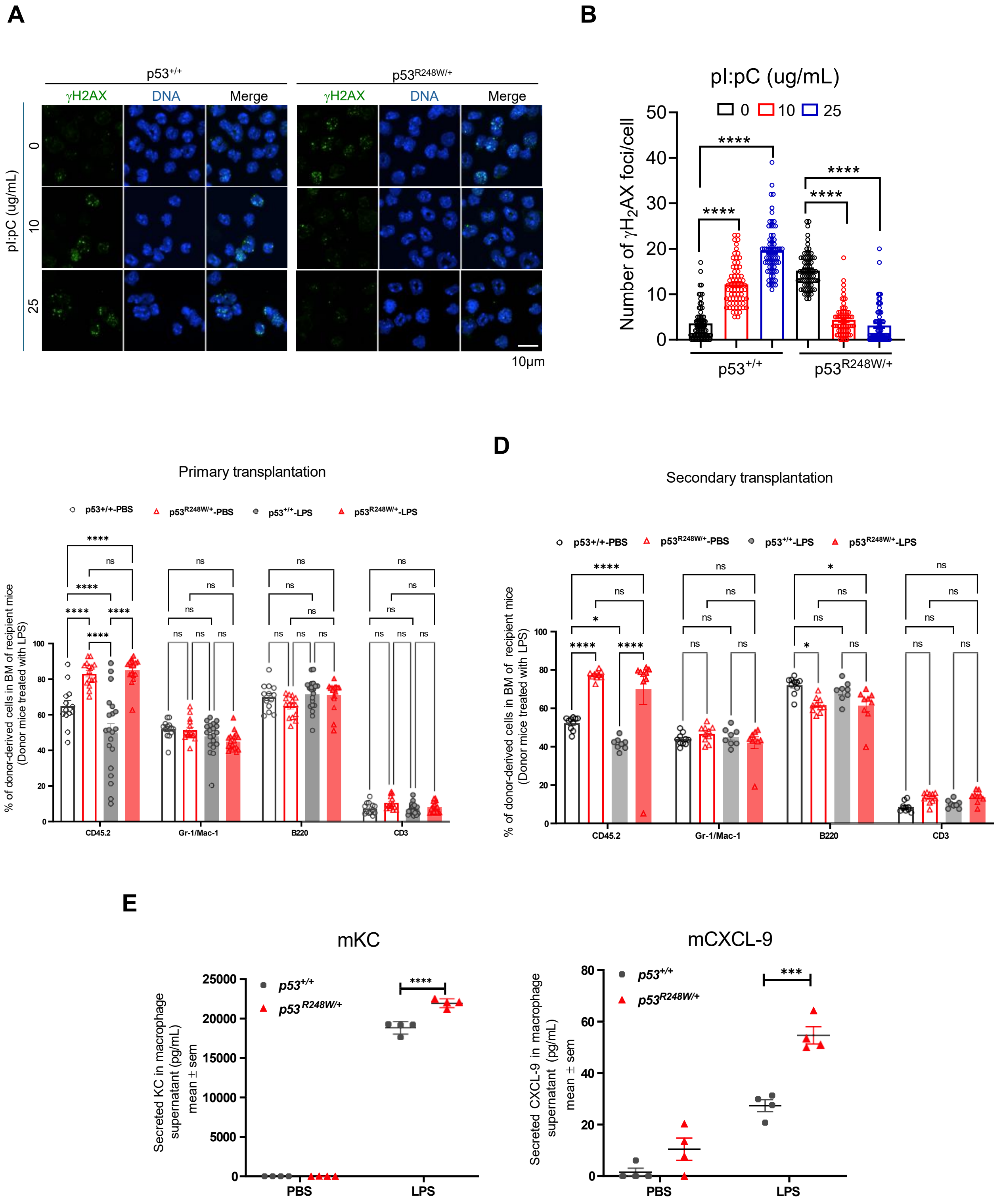

Supplemental Figure 2

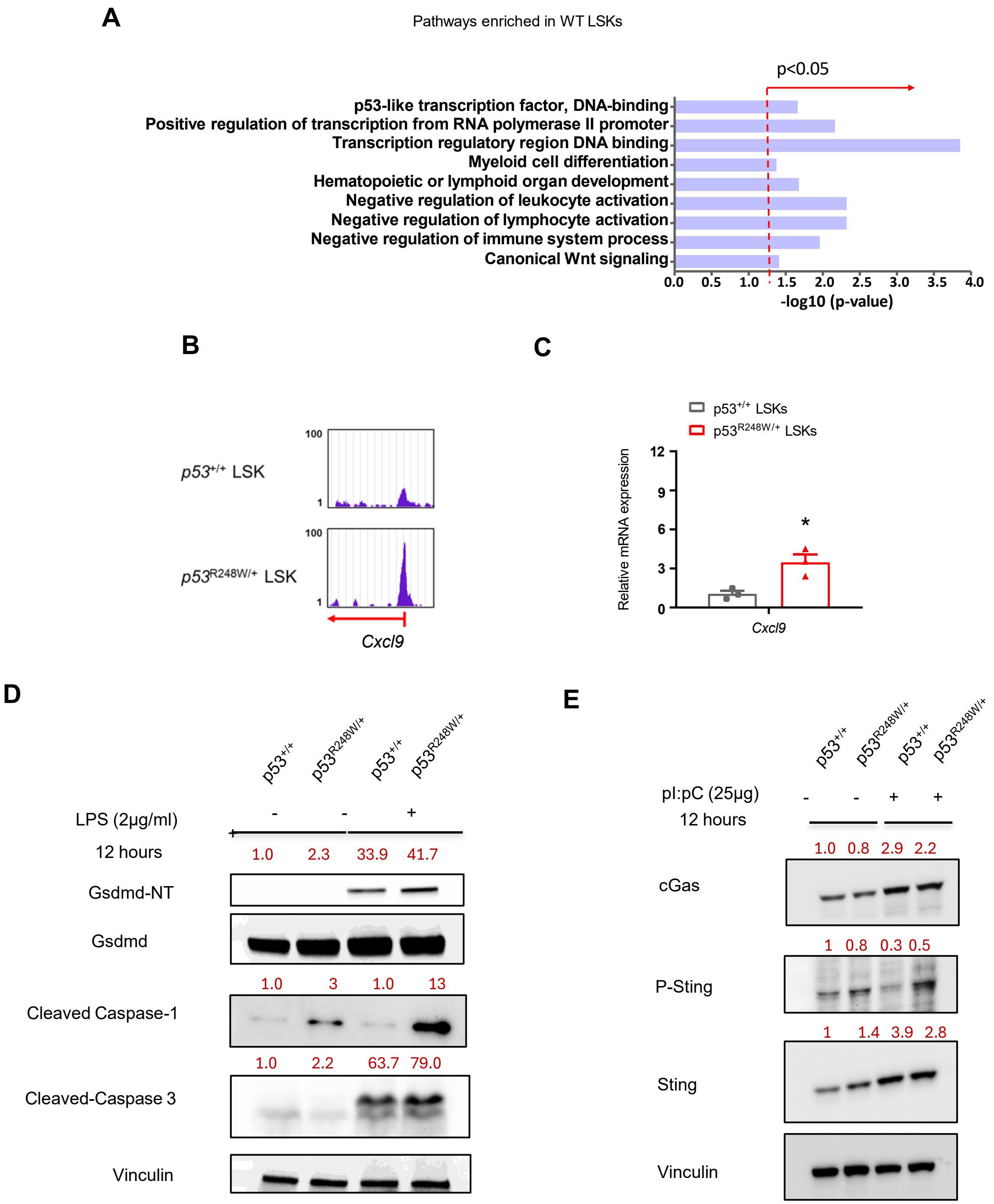

Supplemental Figure 3

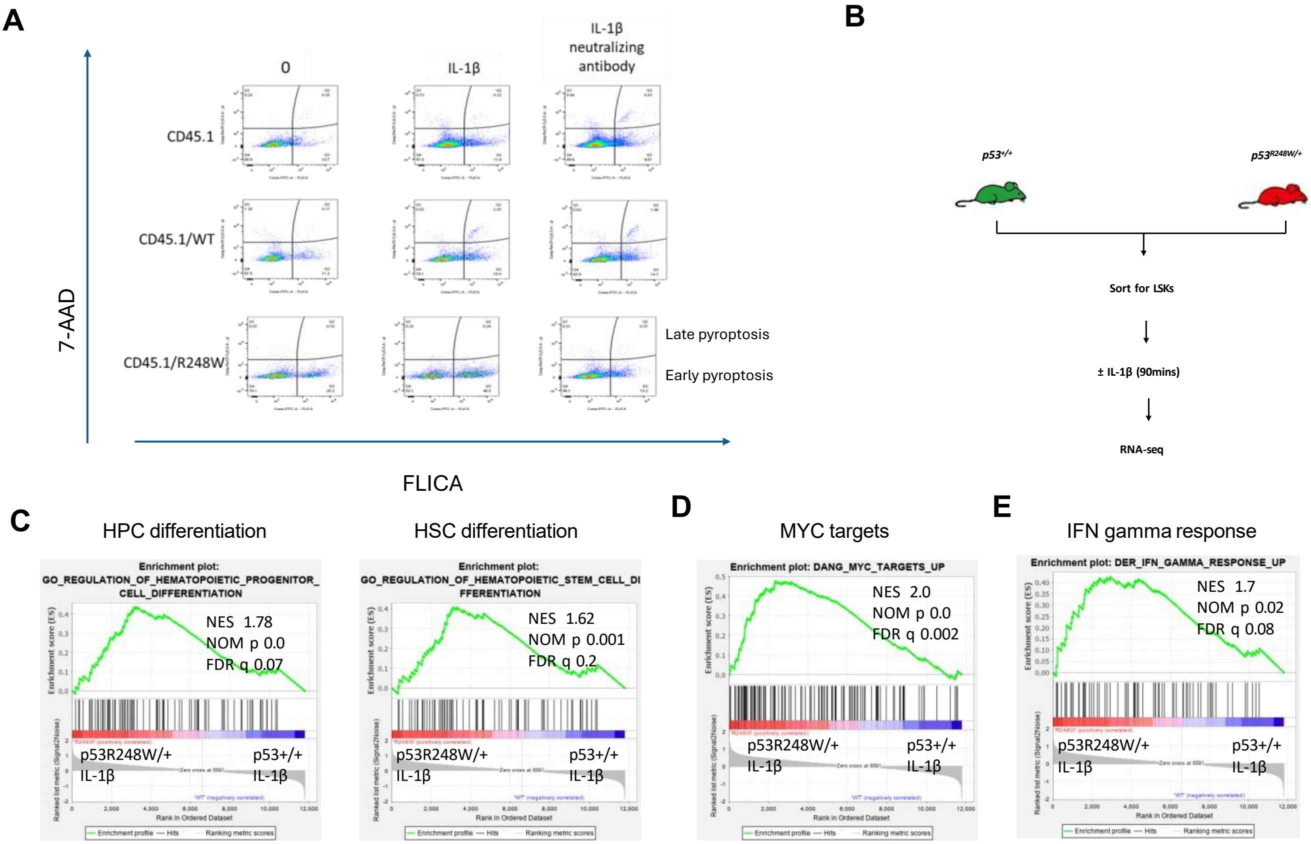

Supplemental Figure 4

A

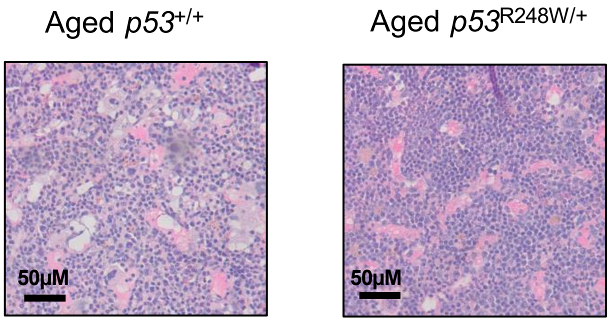

B

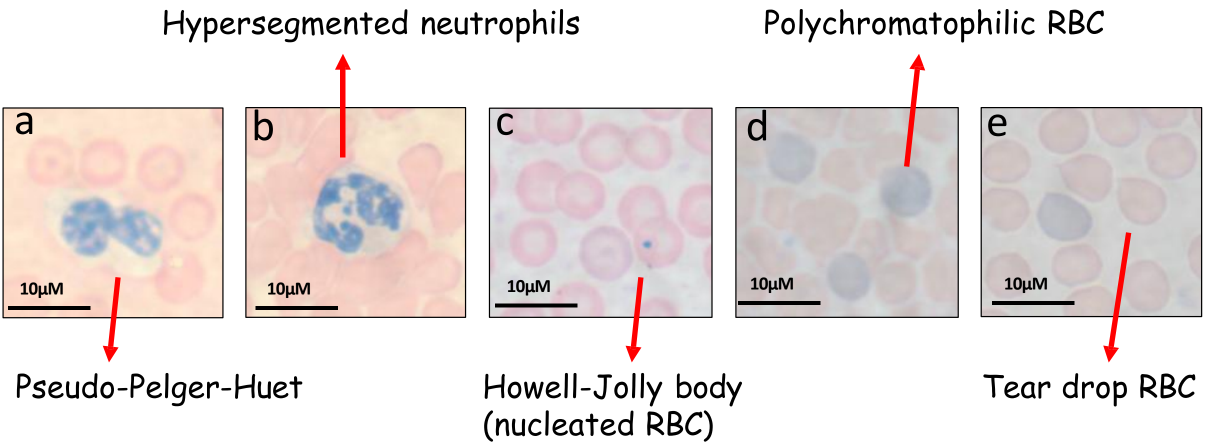

C

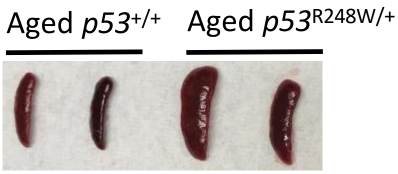

D

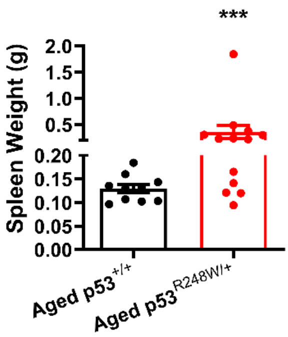

E

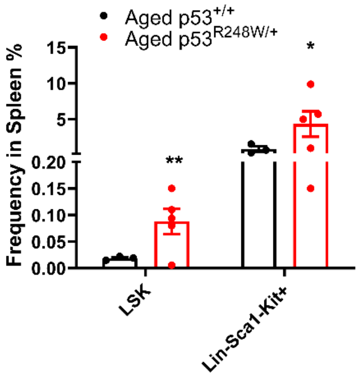

F

Disease Type	Number of Mice
MDS	3
Bone marrow failure	1
Not determined due to death	2

G

SPLICEOSOME

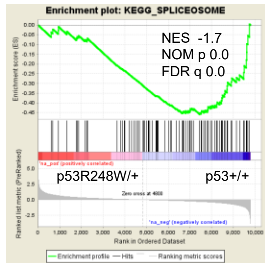

Supplemental Figure 5

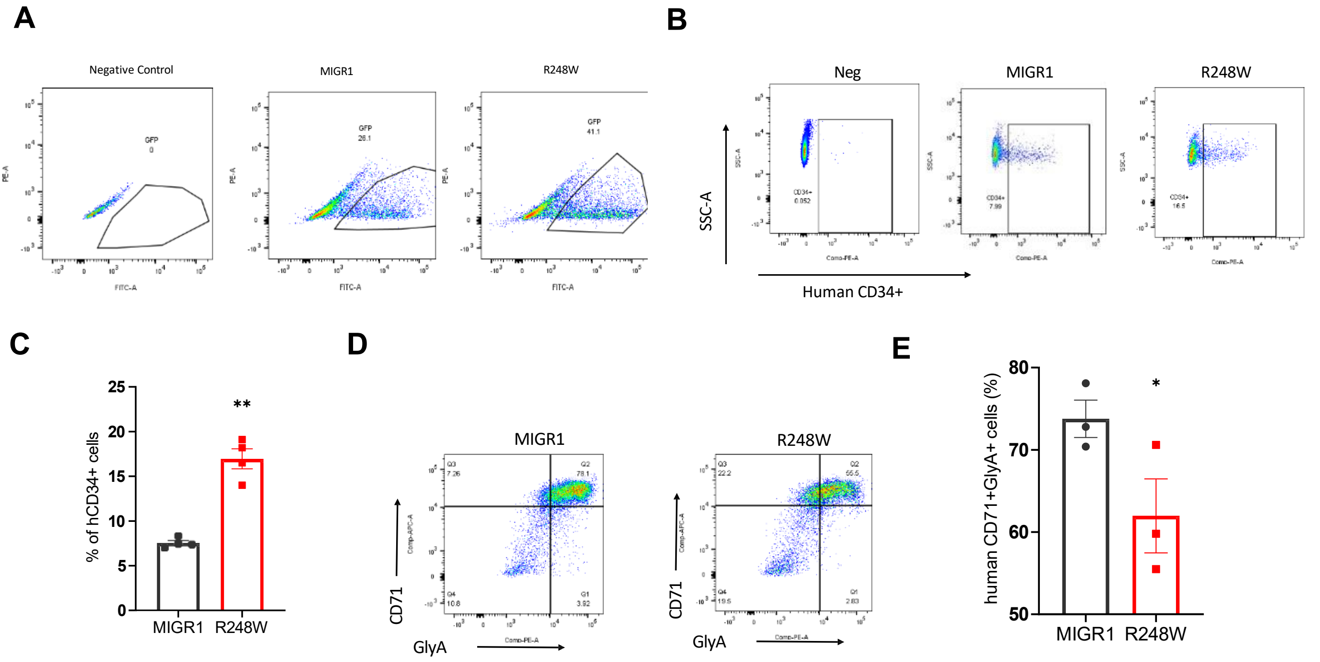

Supplemental Figure 6

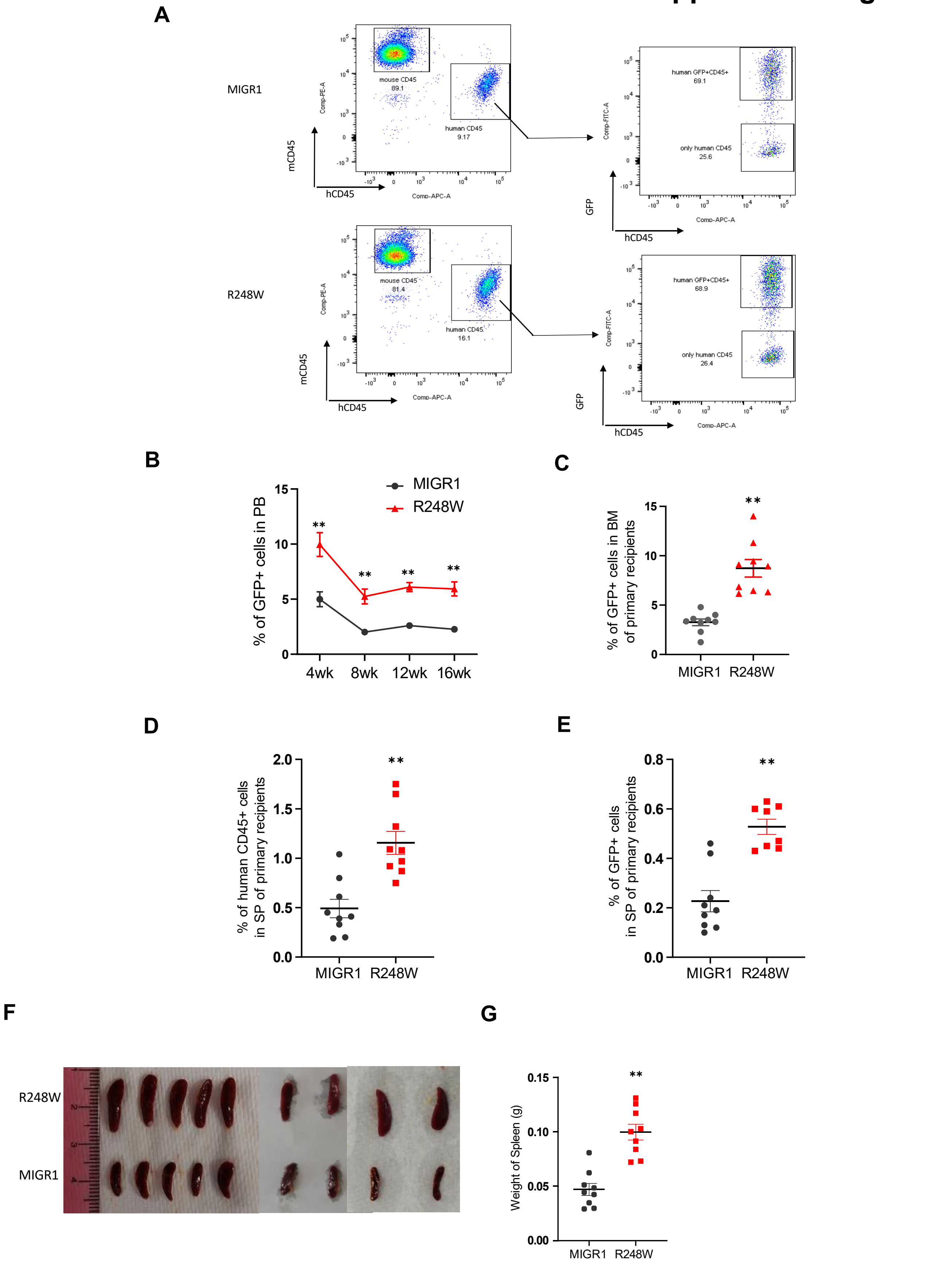

Supplemental Figure 7

A

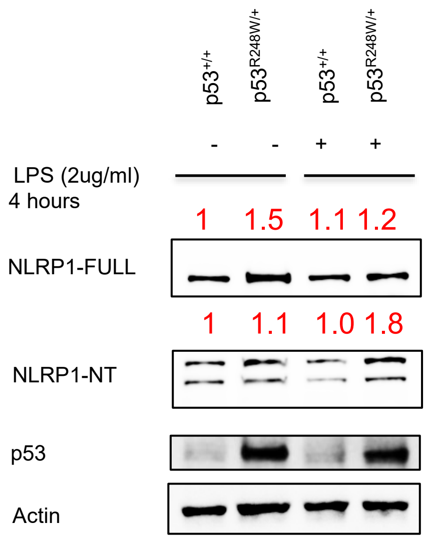

B

CD34 positive MKP

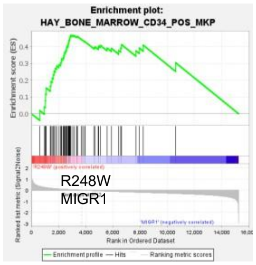

Flow cytometry Antibodies

Antigen/Name	Lot #	Fluorochrome	Company	Catalog #
Chimera analysis				
Combination 1				
CD45.2	B309527	APC	Biolegend	109814
CD45.1	B392799	PE	Biolegend	110708
Combination 2				
CD45.1	B392799	PE	Biolegend	110708
CD45.2	B250186	FITC	Biolegend	109806
Combination 3				
CD45.1	B313623	PB	Biolegend	110722
CD45.2	B309527	APC	Biolegend	109814
HSC analysis				
Lin-Streotavidin	B295808	APCcy7	Biolegend	405208
Sca1	B378036	PB	Biolegend	108120
cKit	B285155	PECy7	Biolegend	105814
CD150	B328902	Percpcy5.5	Biolegend	115922
CD48	B268813	APC	Biolegend	103412

**Western Blot**	Company	Catalog #
**Antigen/Name**		
Phospho-NF-κB p65 (Ser536) (93H1)	Cell signaling	3033S
NF-κB p65 (D14E12) XP®	Cell signaling	8242S
β-Actin (8H10D10)	Cell signaling	3700S
Vinculin (E1E9V) XP®	Cell signaling	13901S
Anti-cleaved N-terminal GSDMD antibody [EPR20829-408]	Abcams	ab215203
Anti-Rabbit IgG	Cell signaling	7074S
Anti-Mouse Igg	Cell signaling	7076S
NLRP3 (D4D8T) Rabbit mAb #15101	Cell signaling	15101S
Cleaved Caspase-1 (Asp296) Antibody #67314	Cell signaling	67314S
Phospho-STING (Ser365) (D8F4W) Rabbit mAb #72971	Cell signaling	72971S
STING (D2P2F) Rabbit mAb #13647	Cell signaling	13647S
cGAS (D3O8O) Rabbit mAb #31659	Cell signaling	31659S
Anti-GSDMD antibody [EPR20859]	Abcams	ab219800
NALP1 Polyclonal Antibody	ThermoFisher	PA5-116672
anti-NLRP1b (mouse), mAb (2A12)	AdipoGen Life Sciences	AG-20B-0084-C100

Used in Cultured	Company	Catalog #
Recombinant anti-mouse IL-1β antibody	Invivogen	mil1b-mab9-02
Recombinant Human IL-1 beta/IL-1F2 Protein	R&D systems	201-LB
