# Supplementary material for: Mutant p53 promotes clonal hematopoiesis by generating a chronic inflammatory microenvironment
Source: J Clin Invest. 2025 Dec 30;136(3):e184285. doi: 10.1172/JCI184285 (PMC12867160; doi:10.1172/JCI184285)
Supplement: Unedited blot and gel images [file jci-136-184285-s332.pdf]

Full unedited blot/gel for Figure 2F

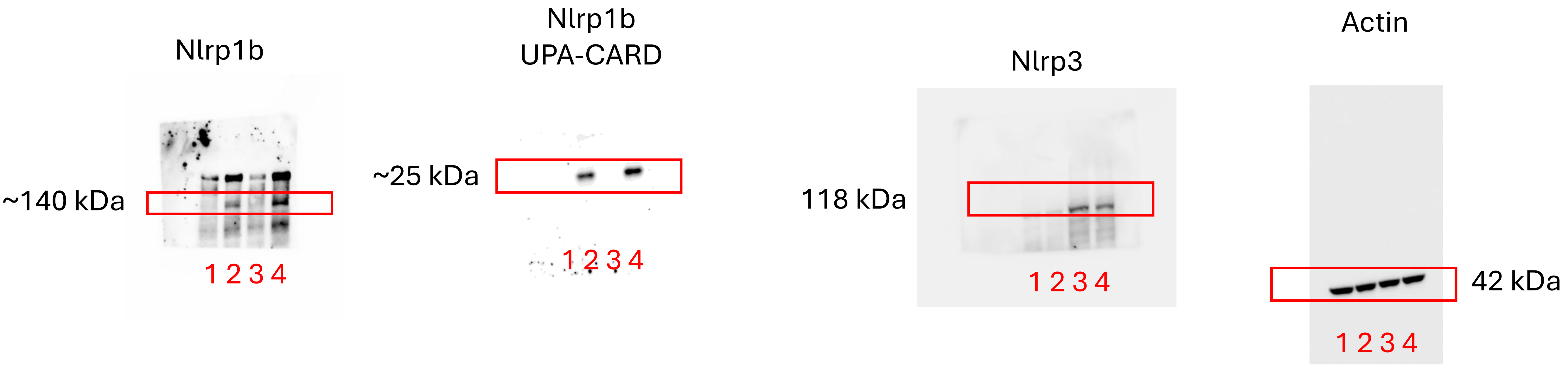

Lane 1: $p53^{+/+}$ Lineage negative BM cells – PBS treated
Lane 2: $p53^{R248W/+}$ Lineage negative BM cells – PBS treated
Lane 3: $p53^{+/+}$ Lineage negative BM cells – LPS (2 ug/ml) treated
Lane 4: $p53^{R248W/+}$ Lineage negative BM cells – LPS (2 ug/ml) treated

Full unedited blot/gel for Figure 2G

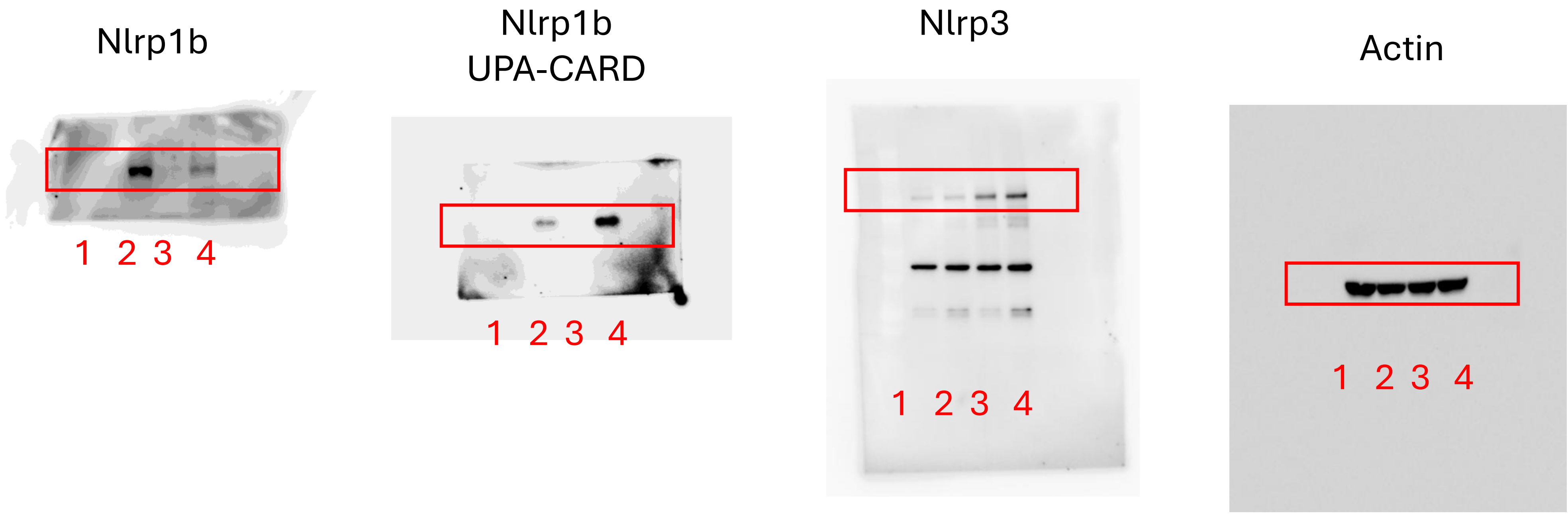

Lane 1: $p53^{+/+}$ Bone marrow derived macrophages – PBS treated
Lane 2: $p53^{R248W/+}$ Bone marrow derived macrophages – PBS treated
Lane 3: $p53^{+/+}$ Bone marrow derived macrophages - LPS (2 ug/ml) treated
Lane 4: $p53^{R248W/+}$ Bone marrow derived macrophages - LPS (2 ug/ml) treated

Full unedited blot/gel for Figure 4F

Gasdermin D (cleaved)

30 kDa

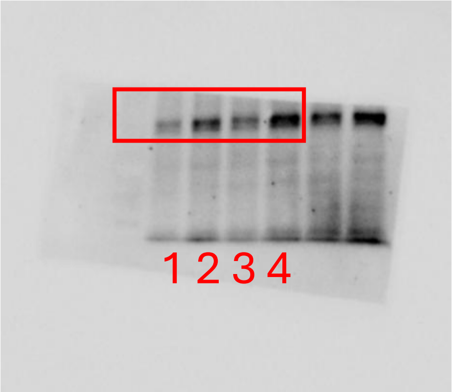

Gasdermin D (full length)

53 kDa

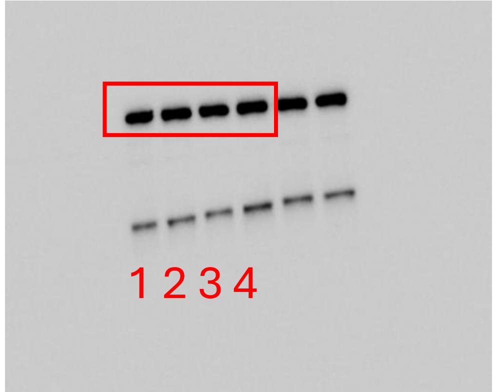

Vinculin

117 kDa

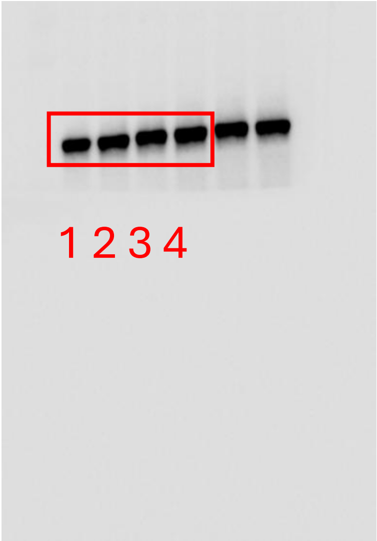

Lane 1: $p53^{+/+}$ Bone marrow mononuclear cells – DMSO treated
Lane 2: $p53^{R248W/+}$ Bone marrow mononuclear cells – DMSO treated
Lane 3: $p53^{+/+}$ Bone marrow mononuclear cells – VbP (10 µM) treated
Lane 4: $p53^{R248W/+}$ Bone marrow mononuclear cells - VbP (10 µM) treated

Full unedited blot/gel for Figure 7D

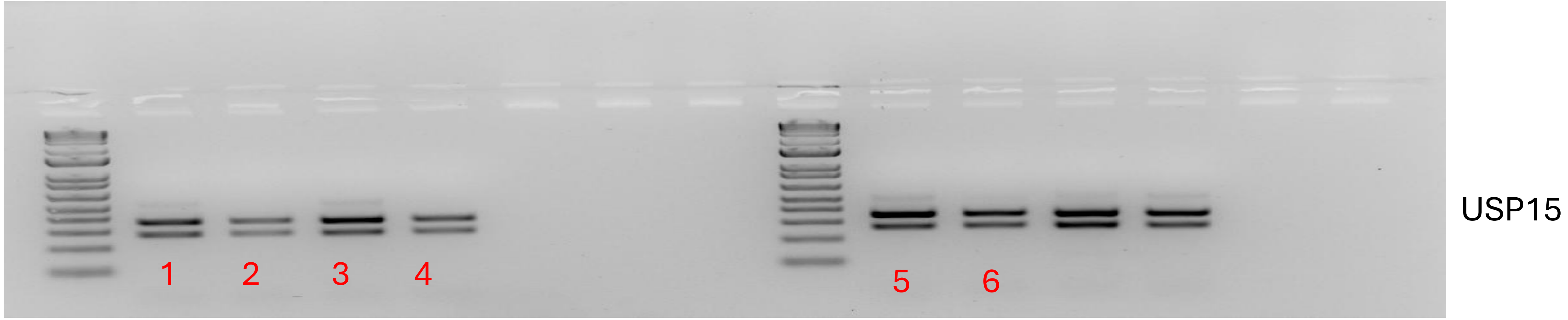

Lane 1: $p53^{+/+}$
Lane 2: $p53^{R248w/+}$
Lane 3: $p53^{+/+}$
Lane 4: $p53^{R248w/+}$
Lane 5: $p53^{+/+}$
Lane 6: $p53^{R248w/+}$

Each one is one biological replicate

Full unedited blot/gel for Figure 7F

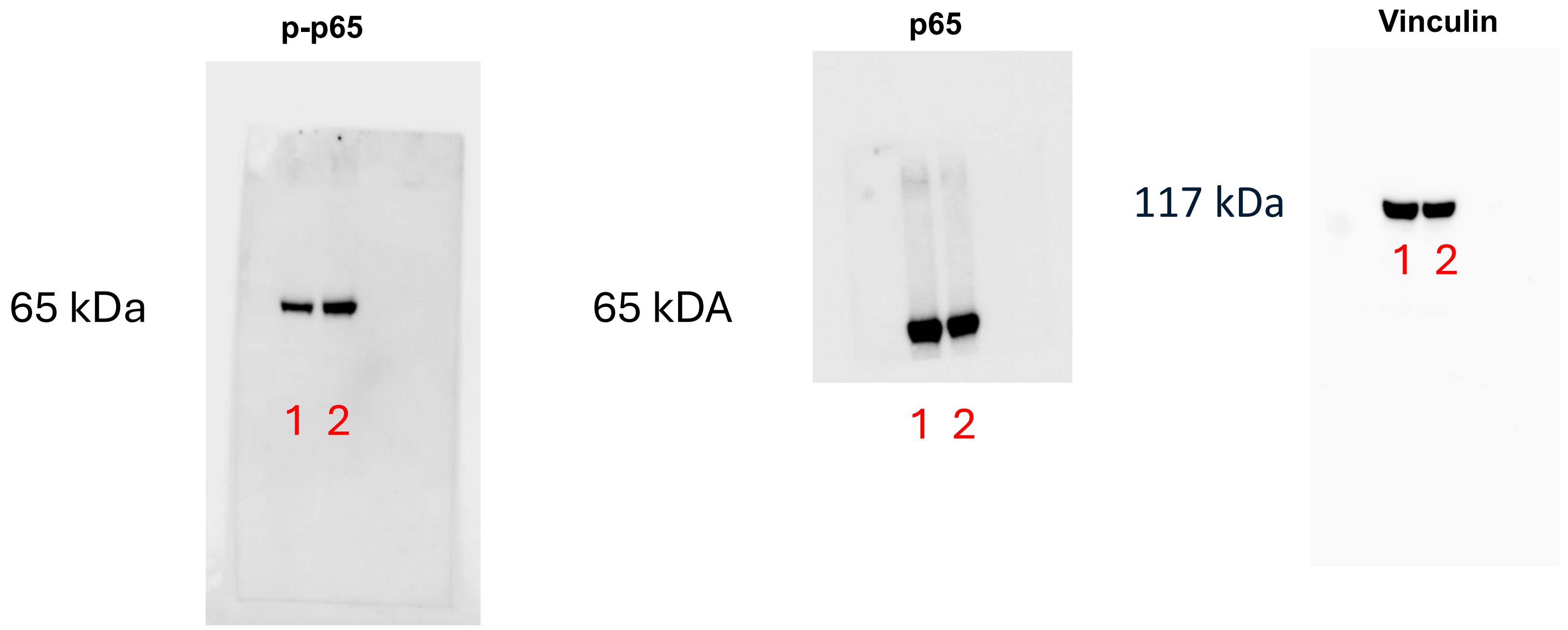

Lane 1: $p53^{+/+}$ Bone marrow mononuclear cells
Lane 2: $p53^{R248W/+}$ Bone marrow mononuclear cells

Full unedited blot/gel for Figure 7G

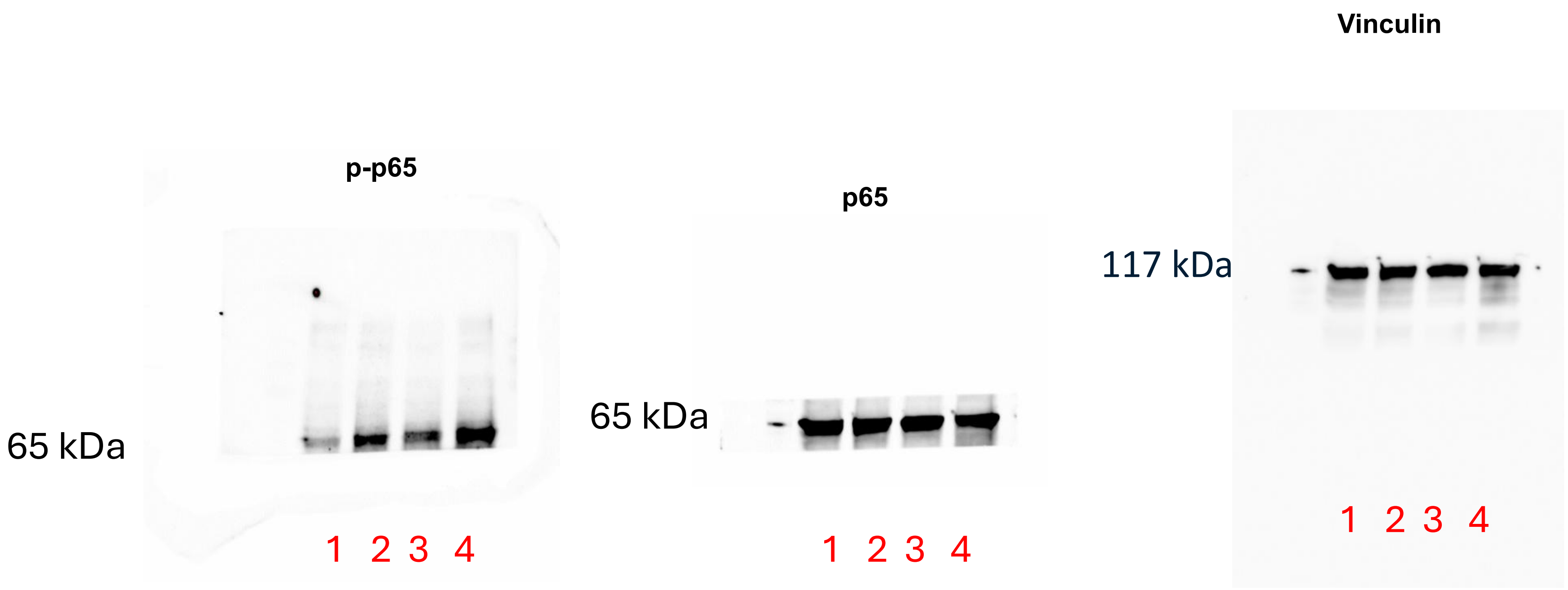

Lane 1: $p53^{+/+}$ Bone marrow derived macrophages – PBS treated
Lane 2: $p53^{R248W/+}$ Bone marrow derived macrophages – PBS treated
Lane 3: $p53^{+/+}$ Bone marrow derived macrophages – LPS (2 ug/ml) treated
Lane 4: $p53^{R248W/+}$ Bone marrow derived macrophages – LPS (2 ug/ml) treated

Full unedited blot/gel for Figure 7H

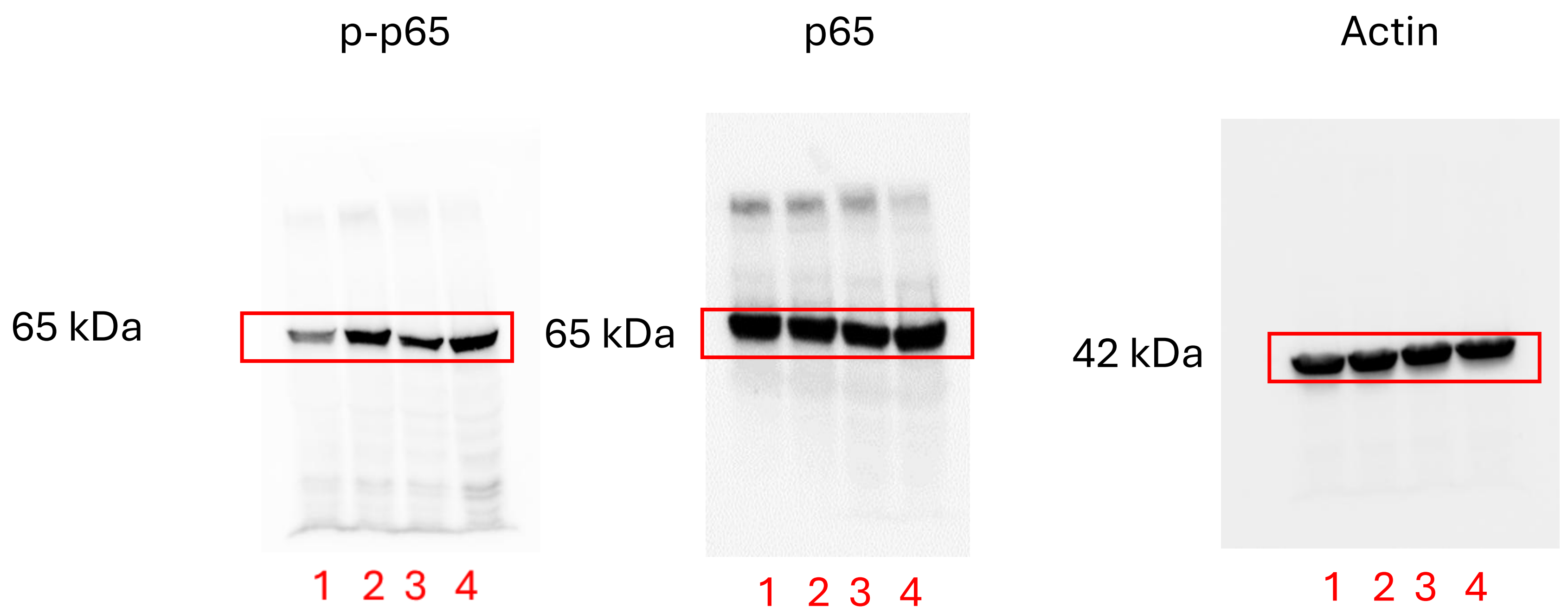

Lane 1: $p53^{+/+}$ Bone marrow derived macrophages – PBS treated
Lane 2: $p53^{R248W/+}$ Bone marrow derived macrophages – PBS treated
Lane 3: $p53^{+/+}$ Bone marrow derived macrophages – LPS (2 ug/ml) treated
Lane 4: $p53^{R248W/+}$ Bone marrow derived macrophages – LPS (2 ug/ml) treated

Full unedited blot/gel for Figure 8K

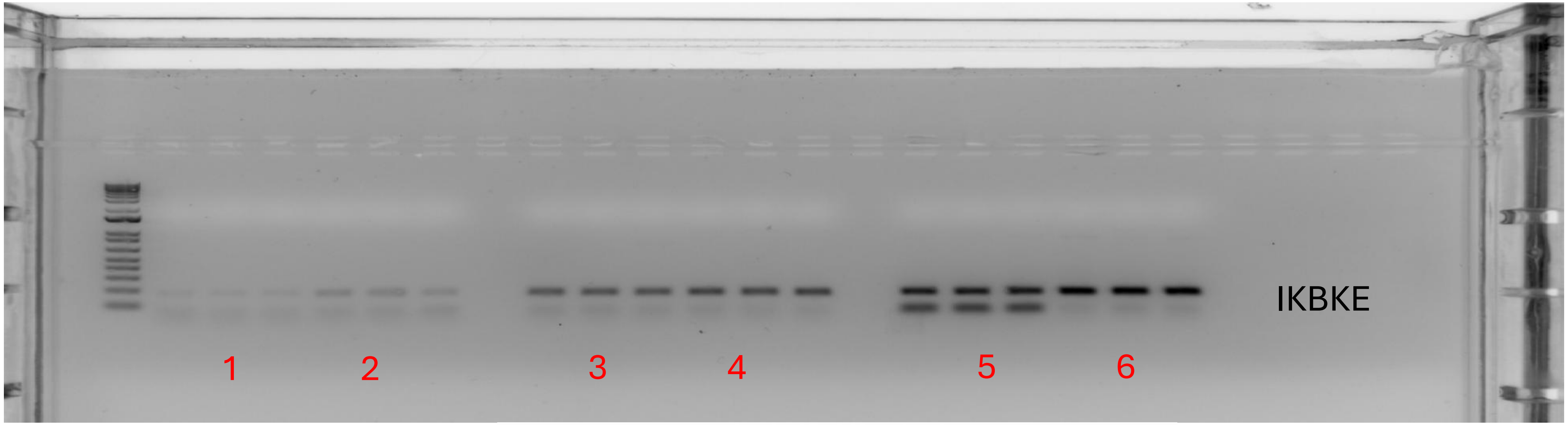

Lane 1: MIGR1
Lane 2: p53^{R248w}
Lane 3: MIGR1
Lane 4: p53^{R248W}
Lane 5: MIGR1
Lane 6: p53^{R248w}
Lane 7: MIGR1
Lane 8: p53^{R248w}

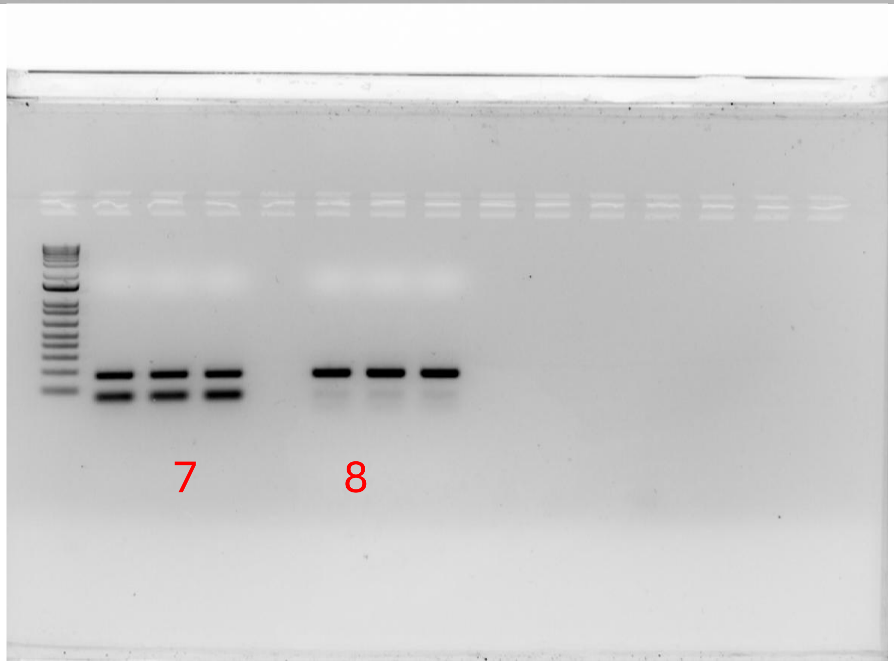

IKBKE

Three technique replicate and three different samples

Full unedited blot/gel for Figure 8M

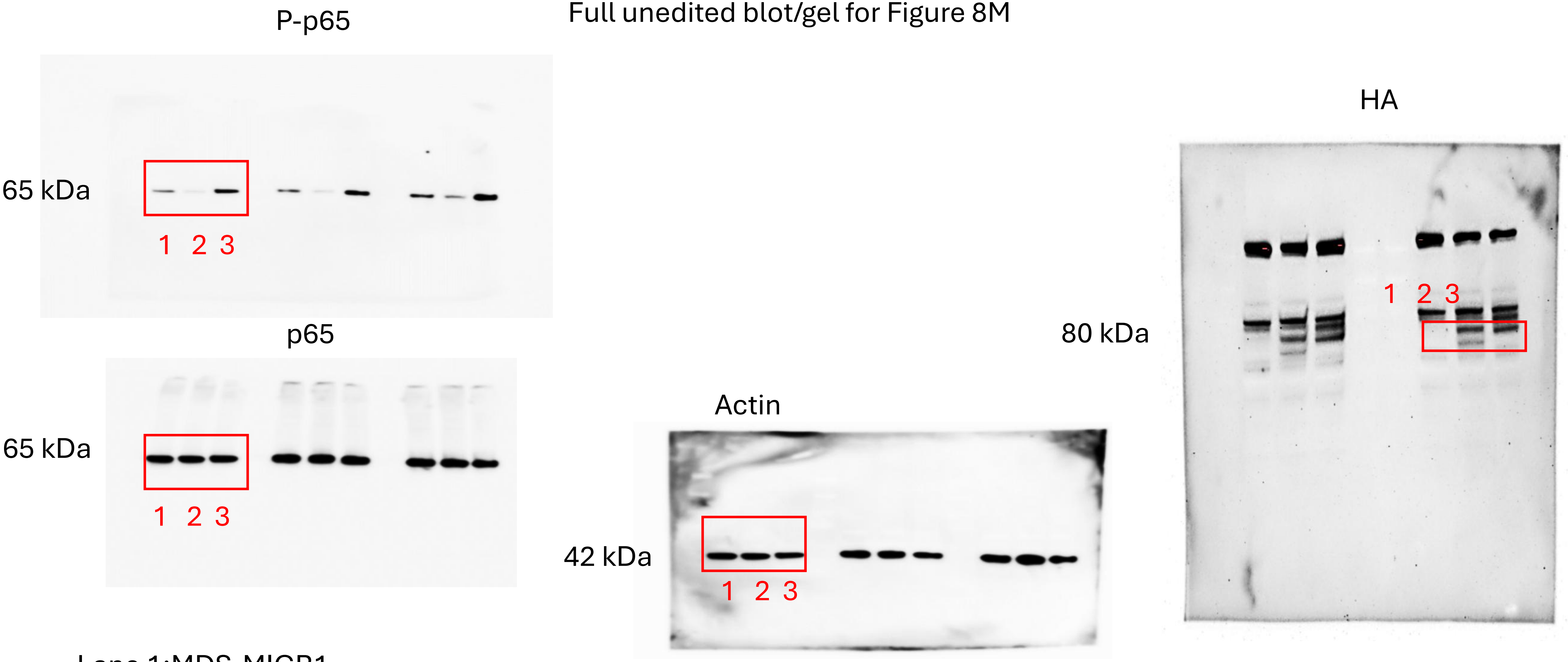

Lane 1:MDS-MIGR1
Lane 2: MDS-IKBKE-Short Isoform
Lane 3: MDS-IKBKE-Long Isoform

Full unedited blot/gel for
Supplemental Figure 2D

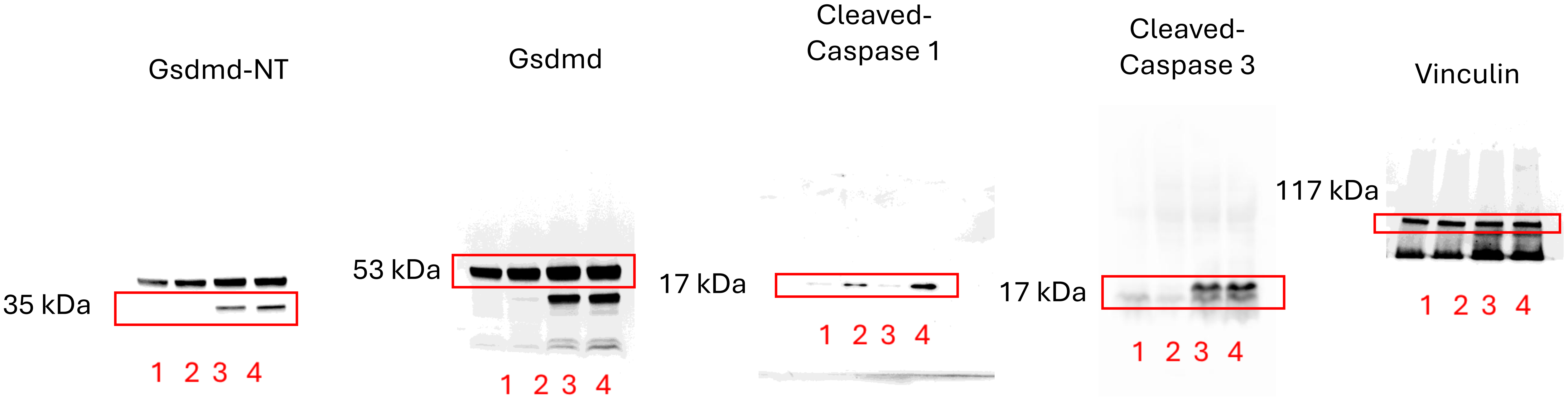

Lane 1: $p53^{+/+}$ Bone marrow derived macrophages – PBS treated
Lane 2: $p53^{R248W/+}$ Bone marrow derived macrophages – PBS treated
Lane 3: $p53^{+/+}$ Bone marrow derived macrophages – LPS (2 ug/ml) treated
Lane 4: $p53^{R248W/+}$ Bone marrow derived macrophages – LPS (2 ug/ml) treated

Full unedited blot/gel for
Supplemental Figure 2E

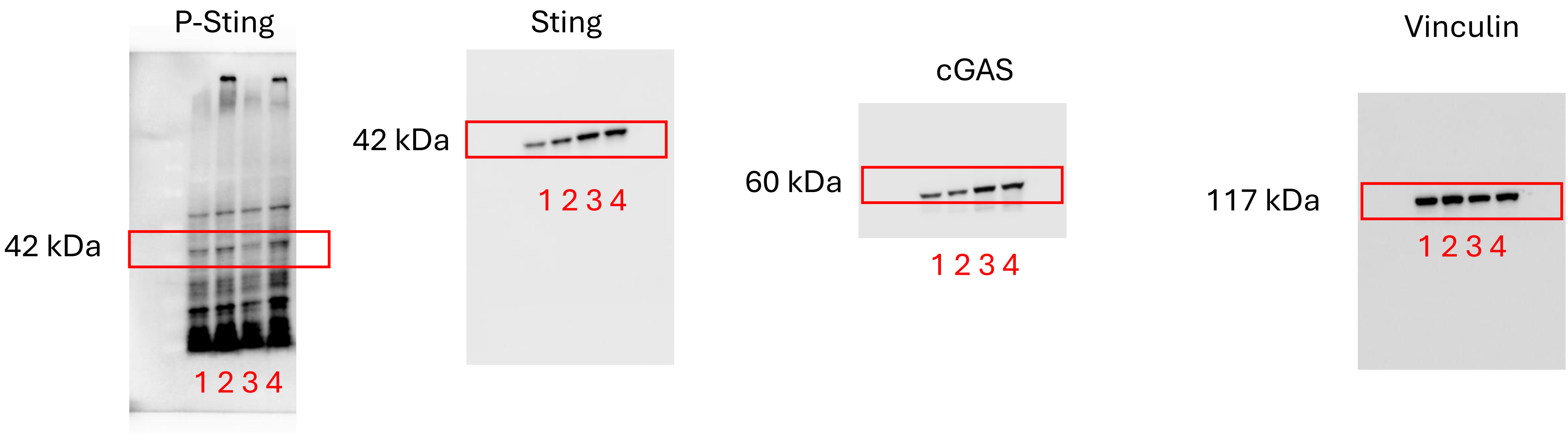

Lane 1: $p53^{+/+}$ Lineage negative cells – PBS treated
Lane 2: $p53^{R248W/+}$ Lineage negative cells – PBS treated
Lane 3: $p53^{+/+}$ Lineage negative cells – pI:pC (25 ug/ml) treated
Lane 4: $p53^{R248W/+}$ Lineage negative cells – pI:pC (25 ug/ml) treated

Full unedited blot/gel for Supplemental Figure 7A

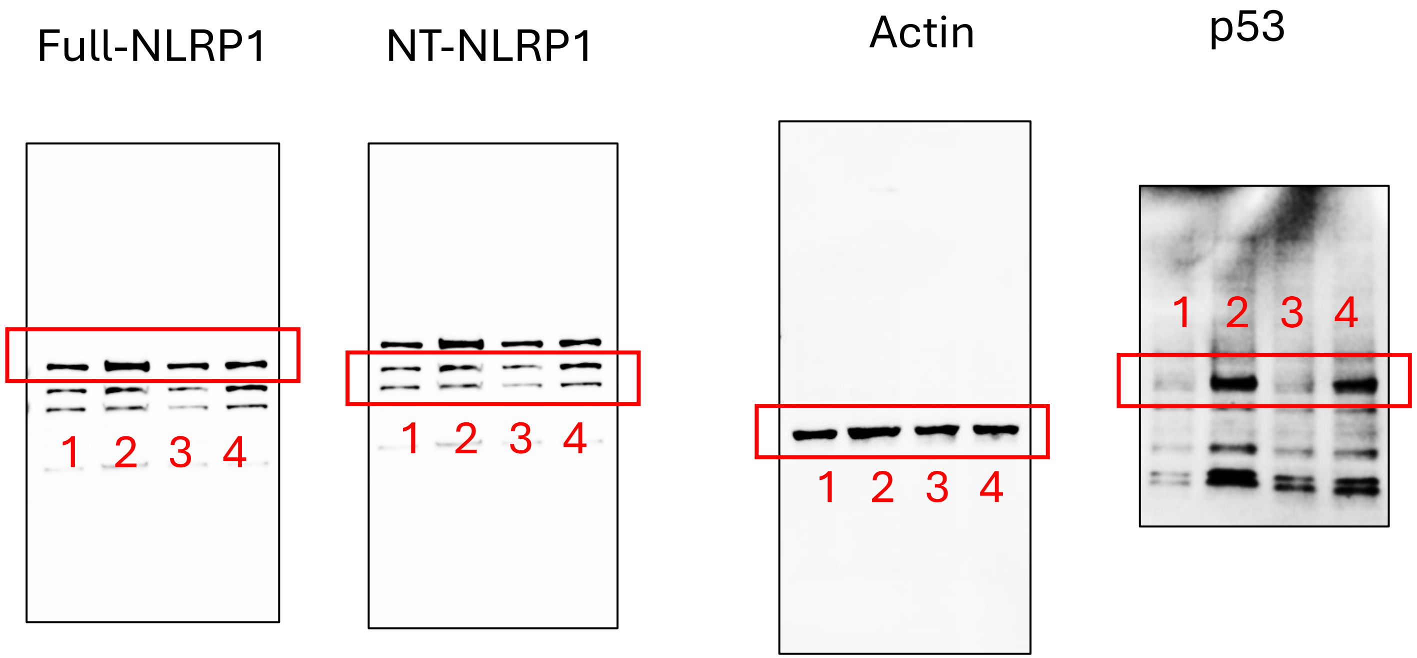

Lane 1: THP1-PMA differentiated Macrophages-MIGR1 – PBS treated
Lane 2: THP1-PMA differentiated Macrophages-R248W - PBS treated
Lane 3: THP1-PMA differentiated Macrophages-MIGR1 – LPS (1 ug/ml) treated
Lane 4: THP1-PMA differentiated Macrophages-R248W – LPS (1 ug/ml) treated
